# Electrochemical Proton-Coupled Electron Transfer Free Energy Surfaces from *Ab Initio* Molecular Dynamics

**DOI:** 10.1021/acs.jpclett.6c02996

**Published:** 2026-10-26

**Authors:** Mahmudul Hasan, Robert E. Warburton

**Affiliations:** Department of Chemical and Biomolecular Engineering, Case Western Reserve University, Cleveland, Ohio 44106, United States

## Abstract

Proton-coupled electron transfer (PCET) is an elementary step in many electrocatalytic reactions, yet molecular-level descriptions of solvent reorganization energetics in these reactions remain limited. Herein, we describe a first-principles computational platform to calculate free energy surfaces and quantify reorganization energies for interfacial electrochemical PCET. Hydrogen electrosorption at Pt(111)/water interfaces is used as a model reaction to derive PCET free energy surfaces using *ab initio* molecular dynamics simulations in an explicit solvent. The reaction is described as a non-adiabatic transition between reactant and product states defined in terms of localization of electrons and the transferring quantized proton. Free energy surfaces are calculated from statistical fluctuations of the vertical energy gap. This computational approach directly incorporates explicit solvent dynamics into the calculated reorganization energies that are obtained from parabolic fits to the reactant and product free energy surfaces. The reorganization energies are quantitatively similar to the mean vertical energy gap near the free energy minima of the reactant and product state free energy surfaces. The calculated PCET reorganization energy of 3.19 eV compares favorably with previous semi-empirical estimates. This work establishes a robust computational platform to assess the role of interfacial solvent properties on intrinsic electrochemical kinetics.

## Graphical abstract

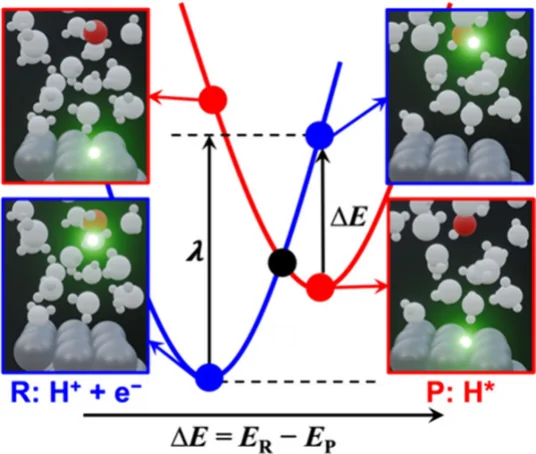

Proton-coupled electron transfer (PCET) is an elementary step common to many electrochemical energy conversion reactions, including hydrogen evolution, oxygen evolution, and carbon dioxide reduction.^1–8^ Theoretical formulations of PCET rate constants are well-established and entail a quantum mechanical treatment of electrons and proton(s) that transfer along a collective solvent coordinate.^7,9–12^ PCET rate constant theory builds off the Marcus theory of electron transfer (ET)^13,14^ and related proton transfer theories,^15^ where the activation free energy is determined by the reaction free energy and reorganization energy.^16^ The reorganization energy corresponds to the energy released due to changes in solute structure and reorientation of the surrounding solvent upon PCET.^17^ The role of solvent reorganization energetics has attracted recent attention given the combined roles that electrode and electrolyte properties have on electrocatalytic rates.^18–23^ For example, experimental studies of the hydrogen evolution reaction (HER) suggest that large interfacial electric fields increase PCET activation energies via higher reorganization energies for the rigid interfacial water present on highly charged electrode surfaces.^16,24–26^ However, the direct influence of electrode properties, electrolyte chemistry, and interfacial electric fields on reorganization energies and activation free energies has not been established due to challenges in evaluating PCET reorganization energies using molecular simulations. Computational frameworks to calculate reorganization energies are needed for a deeper molecular-level understanding of electrochemical PCET reactions across various systems.

Herein, we present a first-principles framework to quantify reorganization energies and activation free energies for electrochemical PCET. Using the Volmer step of HER (i.e., proton electrosorption) at a Pt(111)/water interface as a model reaction, we employ *ab initio* molecular dynamics (AIMD) in explicit solvent to simulate diabatic PCET free energy surfaces from the statistical fluctuations of reactant and product states. The free energy surfaces are used to determine the PCET reorganization energies and activation free energies, which are consistent with previous approximations based on proton solvation energies and static density functional theory (DFT) calculations, demonstrating the ability of the framework to quantitatively describe PCET energetics at electrochemical interfaces with an explicit accounting of solvent dynamics and reorganization effects. This computational platform enables deeper theoretical investigations into the effects of electrode and electrolyte properties on the intrinsic reorganization energies and activation free energies in electrocatalysis.

We adopt a two-state diabatic representation to describe PCET at electrochemical interfaces,^9,10^ analogous to the Marcus theory of ET.^27^ Figure 1 depicts the reactant (R, blue) and product (P, red) diabatic states for the Volmer reaction, a PCET process in which a proton is transferred from the electrolyte to an unoccupied site on the electrode surface (*) where it is reduced by an electron supplied by the electrode to form an adsorbed hydrogen atom, H*. Thus, the R state is defined as H^+^ + e^–^ + * where the transferring proton is in bulk electrolyte, and the P state is defined as H*, where the transferring hydrogen is adsorbed on the electrode surface. Note that in this diabatic representation, the transferring quantized hydrogen is oxidized as a proton in the R state and reduced as an adsorbed hydrogen atom in the P state. The corresponding R and P diabatic free energy surfaces are represented as parabolas along the collective reaction coordinate, as in the linear-response approximations of Marcus theory and the related vibronically non-adiabatic PCET theory. Within these theories, the activation free energy (Δ*A* ^⧧^) is a function of the reaction free energy (Δ*A*) and the reorganization energy (λ).

1 $$\Delta A^{⧧}=\frac{{\left(\Delta A+\lambda \right)}^{2}}{4\lambda }$$

The reorganization energy λ (labeled in Figure 1) corresponds to the free energy difference between equilibrium and non-equilibrium nuclear configurations of the surrounding solvent and interfacial environment within a given diabatic state. Snapshots of representative equilibrium (minima on the Marcus parabolas) and non-equilibrium geometries are shown as insets in Figure 1. The λ is related to the curvature of the free energy surfaces along the reaction coordinate and, along with Δ*A*, determines the activation free energy a key quantity governing reaction kinetics.^7,28–30^ Construction of the R and P free energy surfaces therefore provides a direct route to quantify reorganization energies and predict kinetic barriers for PCET.

**Figure 1:**
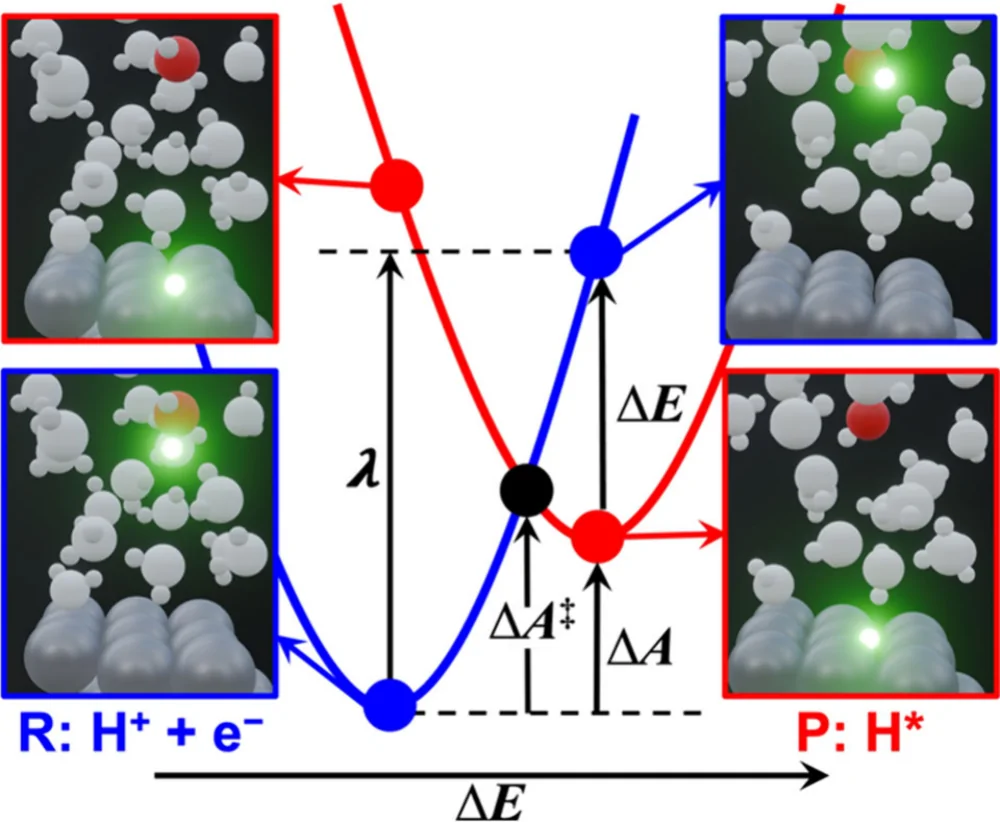
Diabatic representation of the reactant (R, blue) and product (P, red) diabatic states for the Volmer reaction on Pt(111). The parabolas represent the diabatic free energy surfaces along a reaction coordinate defined by the vertical energy gap, Δ*E* = *E*_R_ – *E*_P_. The surfaces are characterized by the reaction free energy Δ*A* and reorganization energy λ, which determine the activation free energy Δ*A* ^⧧^ by eq 1. The framed insets show representative structures for equilibrium (lower frames) and non-equilibrium (upper frames) localization of the quantized hydrogen (bright green).

The vertical energy gap (Δ*E*) is a convenient representation of the multidimensional reaction coordinate.^31^ Δ*E* is defined as the instantaneous energy difference between two diabatic states evaluated at a fixed nuclear configuration (**r** ^*N*^) of a *N* atom system.

2 $$\Delta E\left(r^{N}\right)=E_{R}\left(r^{N}\right)-E_{P}\left(r^{N}\right)$$

This energy splitting is labeled on the PCET free energy curves in Figure 1, where *E*_R_(**r** ^*N*^) and *E*_P_(**r** ^*N*^) are the potential energy of R and P states at a given ***r*** ^N^, respectively. Warshel showed that Δ*E* fluctuations contain the necessary statistical information to derive diabatic free energy surfaces and, in turn, determine reaction free energies, reorganization energies, and activation free energies.^32^ Sprik and co-workers extended this approach to electrochemical ET, and suggested that AIMD affords more accurate descriptions of electronic structure and bond breaking/forming energetics.^33–36^ This general approach has been applied to the study of various outer-sphere ET and molecular PCET reactions using classical and *ab initio* MD.^37–46^ Given the transferability of these approaches to various homogeneous ET and PCET systems, we posit that this framework can be adapted to diabatic representations of heterogeneous electrochemical PCET.

Diabatic free energy surfaces can be determined from statistical fluctuations of Δ*E*. Within the linear-response approximation of Marcus theory, the probability distribution function of Δ*E* in each diabatic state *M* [*P*_*M*_(Δ*E*)] follows Gaussian statistics

3 $$P_{M}\left(\Delta E\right)=\frac{1}{\sqrt{2\pi {\sigma_{M}}^{2}}}\exp\left[-\frac{{\left(\Delta E-{\left\langle \Delta E\right\rangle }_{M}\right)}^{2}}{2{\sigma_{M}}^{2}}\right]$$

where ⟨Δ*E*⟩_*M*_ and σ_*M*_ are the average and standard deviation of Δ*E* in state *M*, respectively. Note that ⟨Δ*E*⟩_*M*_ corresponds to the energy gap coordinate where the free energy for state *M* is minimized. The corresponding Helmholtz free energy curves are calculated using

4 $$A_{M}\left(\Delta E\right)=-k_{B}T\ln\left[P_{M}\left(\Delta E\right)\right]$$

where *k*_B_ is the Boltzmann constant and *T* is the temperature. The free energy difference between the two diabatic states can be approximated using the relation

5 $$A_{R}\left(\Delta E\right)-A_{P}\left(\Delta E\right)=\Delta E$$

which has several useful implications.^47^ For instance, the non-equilibrium free energy of one state can be obtained from the equilibrium free energy of the other state (from eq 4) and Δ*E*. For Gaussian probability distributions with the same energy gap variance in each state (i.e., σ_R_^2^ = σ_P_^2^), the above equations imply that the reaction free energy Δ*A* and reorganization energy λ can be calculated from the average Δ*E* from each trajectory; i.e., the energy gap at the minimum of each Marcus parabola.

6 $$\Delta A=\frac{1}{2}\left({\left\langle \Delta E\right\rangle }_{P}+{\left\langle \Delta E\right\rangle }_{R}\right)$$

7 $$\lambda =\frac{1}{2}\left({\left\langle \Delta E\right\rangle }_{P}-{\left\langle \Delta E\right\rangle }_{R}\right)$$

Note that λ corresponds to the total reorganization energy, which consists of both inner- and outer-sphere contributions. These expressions can be used to obtain the activation free energy using the familiar Marcus expression in eq 1.

We used AIMD to simulate the dynamics of the reactant and product diabatic states of the Volmer reaction at Pt(111)/water interfaces. All DFT calculations were performed with Quantum ESPRESSO^48,49^ using the PBE exchange–correlation functional with D3 dispersion.^50,51^ *NVT* AIMD simulations were performed at 350 K using deuterated water and a 1 fs time step under Langevin,^52^ Berendsen,^53^ and Nosé–Hoover^54^ thermostats. Various thermostats were tested given the tendency for vibrational energy to shift to translational energy during simulations of metal–water interfaces with Berendsen and Nosé–Hoover thermostats; i.e., the “flying ice cube effect”.^55^ Additional computational details are provided in the Supporting Information.

The AIMD simulations reproduce the structural and dynamical properties of the Pt(111)/water interface. The calculated radial distribution functions, interfacial water density profiles, and vibrational density of states are summarized in Figures S5–S9. These analyses suggest that all three thermostats reproduce properties of interfacial water, yielding the expected bilayer structure on Pt(111) and a calculated vibrational spectra consistent with previous measurements and simulations of deuterated water.^56–59^ Consistent with previous reports,^55^ however, the kinetic energy partitioning between vibrational, rotational, and translational degrees of freedom is thermostat dependent (Figure S10 and Tables S1 and S2). The Berendsen thermostat incorrectly transfers kinetic energy from vibrational degrees of freedom to translational degrees of freedom, consistent with a flying ice cube effect.^55^ In contrast, Nosé–Hoover dynamics with a self-consistency (SCF) threshold of 10^–7^ Ry and Langevin dynamics with a threshold of 10^–4^ Ry accurately reproduce the expected equipartition of energy and yield statistically equivalent structural and dynamical properties. These Nosé–Hoover and Langevin thermostat settings were adopted for the subsequent Δ*E* analysis.

The statistical distributions of Δ*E* were obtained from sampling AIMD trajectories of the reactant and product states. Following a 10 ps equilibration, Δ*E* sampling was performed using snapshots taken at 40 fs intervals consistent with sampling intervals used in previous ET studies.^60,61^ Δ*E* is defined as the energy difference between the two diabatic states given the same set of nuclear coordinates **r** ^*N*^. Because the transferring proton is treated quantum mechanically within in the diabatic representation of the PCET reaction analyzed herein, the classical position of the hydrogen nucleus is not included in the definition of **r** ^*N*^. In practice, the Δ*E* is computed at each snapshot as the energy difference between two calculations: (1) a single-point energy calculation of the hydrogen in its equilibrated position from the AIMD trajectory of either the R or P state, (2) a partial geometry optimization of only the quantized hydrogen in its non-equilibrium configuration (i.e., either in bulk solution or adsorbed on the surface) for fixed geometries of all other nuclei. This is depicted schematically in Figure 1 by the stacked insets on the left and right sides of the PCET Marcus parabola, where only the quantized hydrogen (bright green) moves between the surface and bulk solution for Δ*E* calculations.^62^ This proton-only optimization procedure ensures that proton coordinate reorganization is excluded from the Δ*E* calculation and has been used in previous theoretical PCET studies.^43,46,63^ Note that as depicted in Figure 1, the proton donor and acceptor is chosen to be a water molecule in the bulk water region. We therefore note that the stoichiometry is different between the calculations derived from the reactant and product AIMD trajectories; however, the stoichiometry is maintained when comparing energetics in Δ*E* calculations (section S1.3 of the Supporting Information). Water molecules were chosen in this region of the simulation cell given the quantum mechanical effects of delocalized charged defects in water chains associated with Grotthuss proton transport.^64–67^ In turn, this choice of proton donor/acceptor accounts for reorganization energetics associated with both proton electrosorption on the electrode surface as well as proton relay between the bulk electrolyte and interface. Further details on this procedure are provided in section S5 of the Supporting Information.

The reactant and product potential energies were calculated using DFT for each snapshot along the Langevin AIMD trajectories (Figure 2) and Δ*E* was calculated using eq 2 (Figure 2A and B). Similar insights are obtained from the Nosé–Hoover trajectories (Figure S15). The histograms shown in Figure 2C and D show the distribution of the Δ*E* values computed along the reactant and product trajectories, respectively. We fit a Gaussian distribution to these data to conforms to the underlying linear-response approximation of Marcus theory. Though we assumed Gaussian distribution of Δ*E* for all cases to align linear-response, Anderson–Darling tests for normality suggest that the product states deviate from the Gaussian distribution (Table S3). The deviations may be due to sampling limitations or nonlinear solvent response. While longer simulations would enable further sampling more rigorous statistics,^68^ the calculated reorganization energies are invariant beyond 5 ps (Figure S14). In turn, the *P*_*M*_(Δ*E*) are fit to a Gaussian distribution, and the fitting details are given in Table S3.

**Figure 2:**
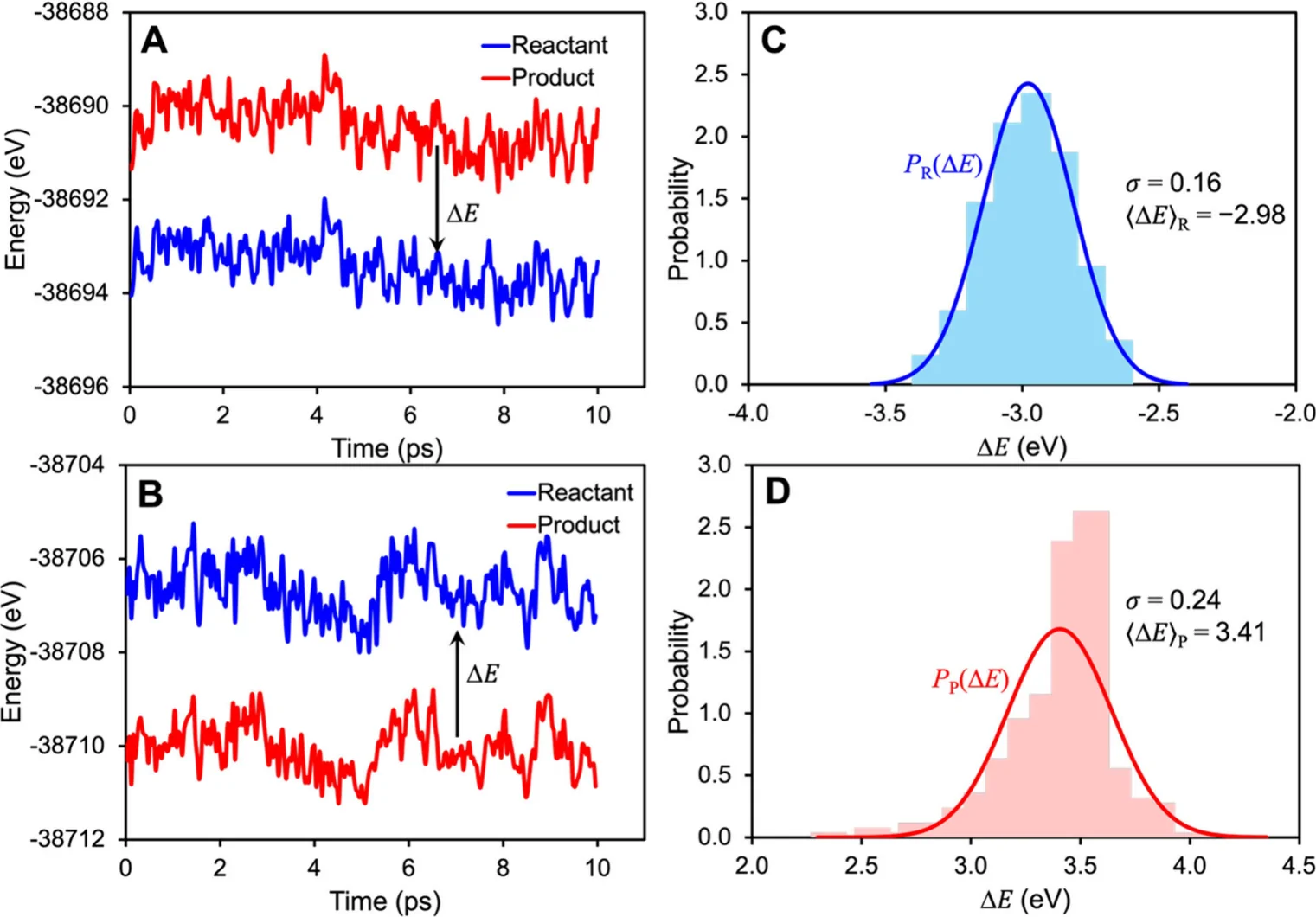
(A and B) DFT energies along the AIMD trajectories of the (A) reactant and (B) product state. The low energy curves are obtained directly from the AIMD simulations and the high energy curves are computed based on the instantaneous vertical energy gap (Δ*E*) for the associated snapshot. (C and D) Calculated distribution of (Δ*E*) for (C) reactant and (D) product states. The probability distribution functions *P*_*M*_(Δ*E*) are fit to a Gaussian distributions with mean ⟨*E*⟩_*M*_ and standard deviation σ_*M*_.

The diabatic free energy surfaces (Figure 3) are fit to a quadratic using free energies and energy gaps determined from the probability distributions *P*_*M*_(Δ*E*) shown in Figure 2C and D. The expression in eq 4 is used to calculate the equilibrium free energies, represented by the data points near the minima of the free energy surfaces in Figure 3. The non-equilibrium free energies are calculated using the eq 5, which are shown by the higher energy data points in Figure 3.^34,69^ These two sets of data points are subject to a parabolic fit (solid lines in Figure 3) to conform to the underlying approximations of Marcus and PCET theory. The energy gap distributions and free energy curves are also used to obtain the PCET reorganization energy and activation free energy using eqs 7 and 1, respectively. Because electrochemical PCET is a faradaic reaction, the reaction free energy is a function of applied potential. At thermoneutral conditions where the reaction free energy for the Volmer reaction is Δ*A* = 0 (i.e., the standard-state reversible potential for the elementary step), the activation free energy depends purely on the reorganization energy, Δ*A* ^⧧^ = λ/4. Here, we calculate a PCET reorganization energy of 3.19 eV, corresponding to a thermoneutral activation free energy of 0.80 eV. Analysis of the Nosé–Hoover trajectories yields a similar reorganization energy of 3.23 eV (Figure S16). The computed reorganization energies from the fitted Marcus parabolas are quantitatively similar to those calculated from the average of the two energy gap distributions using eq 7, where the latter approach yields reorganization energies of 3.20 and 3.23 eV from the Langevin and Nosé–Hoover trajectories, respectively. In principle, the reorganization energy can also be calculated from the width of the Δ*E* distribution using λ = σ^2^/2*k*_B_ *T*; however, this relationship yields much lower reorganization energies of 0.42 and 0.96 eV for the R and P states, respectively, which is inconsistent with values obtained from the free energy curves and eq 7. Under the Marcus linear-response approximation, all three values should be equivalent; however, we note that such discrepancies have also been observed in several other ET studies which accounts for discrepancies between the data points and the fitted quadratics.^34,60,61,69^ Although we find that Gaussian fit parameters are invariant beyond 5 ps (Figure S14), these discrepancies may reflect limited configurational sampling of infrequent solvent configurations that would increase the variance of *P*_*M*_(Δ*E*). Future studies could incorporate machine learning interatomic potentials, for example, which could improve sampling statistics while maintaining near-DFT accuracy.^70–73^

**Figure 3:**
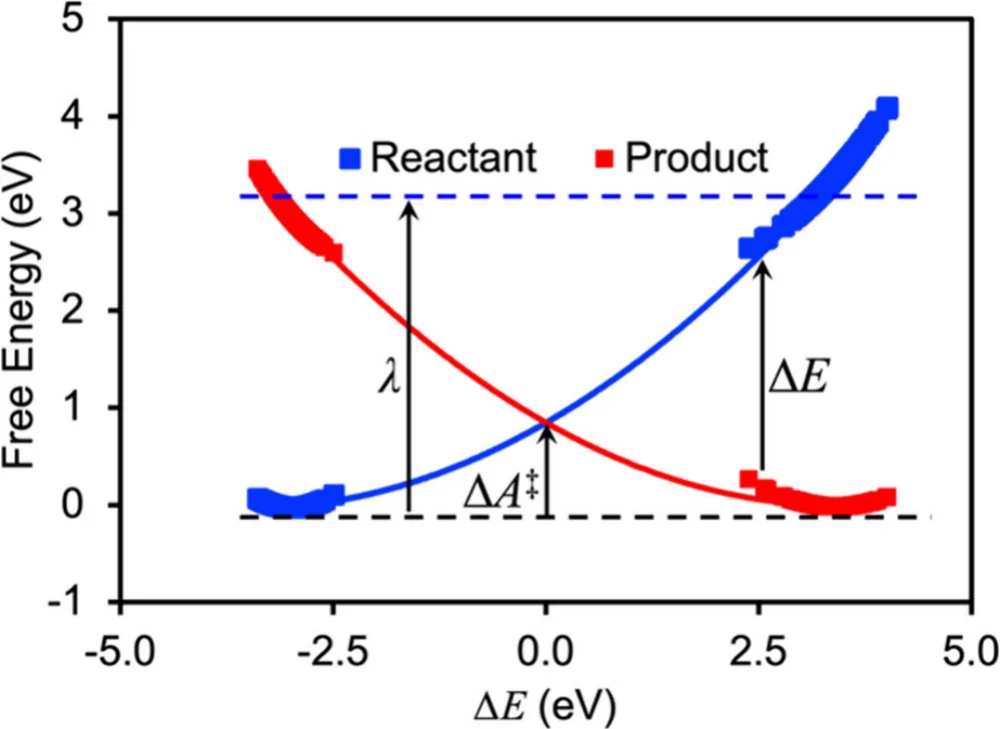
Diabatic PCET free energy surfaces as a function of Δ*E* for the Volmer reaction at the Pt(111)/water interface. Free energy curves are fit using Marcus parabolas in accordance with the assumptions underlying linear-response PCET theory.

The calculated PCET reorganization energy (3.19 eV) and thermoneutral activation free energy (0.80 eV) fall within a range of quantities reported in previous computational studies that employed different modeling strategies.^74–79^ Our model accounts for fluctuations in solvent reorganization energetics for the net process of proton relays via Grotthuss transport from the bulk solvent and electrosorption to the Pt(111) surface. The free energies of the reactant and product states are modeled within the weak vibronically adiabatic approximation such that electron and proton density does not equilibrate along the collective solvent coordinate for PCET. Differences between our modeling approach and previous studies yield computed values that are either larger or smaller than those from previous reports. For example, the calculated activation free energy is larger than those obtained with DFT nudged elastic band calculations of the adiabatic minimum energy path for PCET from a water molecule near the electrode surface.^76^ In contrast, recent classical simulations reported a higher reorganization energy of 5.5 eV.^78^ The PCET reorganization energies calculated herein are similar to previous estimates of 3–4 eV based on the proton solvation energy.^74,75^ Ideally, computed values should be compared to experimental measurement for further benchmarking and validation of different modeling approaches. Activation energies have been measured for hydrogen evolution and oxidation.^80–84^ However, the potential-dependent activation energies measured experimentally correspond to apparent kinetic quantities for the overall reaction rather than a single elementary step, which makes the direct comparisons with computed barriers challenging.^85,86^ The relative consistency between the model employed in this work and previous modeling studies suggests that the modeling platform devised herein establishes a quantitative *ab initio* framework to account for solvent dynamics in the simulations of diabatic free energy surfaces, reorganization energy, and activation free energies for PCET reactions at electrochemical interfaces.

We have demonstrated a computational framework to quantify interfacial PCET reorganization energies and activation free energies from molecular simulations. Starting from a diabatic representation of the Volmer step of the hydrogen evolution reaction at a Pt(111)/water interface, we derived PCET free energy surfaces from fluctuations in the vertical energy gap between reactant and product diabatic states sampled from AIMD simulations. The quantitative agreement between our results and prior theoretical estimates of PCET reorganization energies highlights the utility of this approach for kinetic analyses of electrochemical PCET. This work provides a useful computational platform to establish connections between interfacial structure, dynamics, and electric fields on PCET reorganization energies and intrinsic electrochemical kinetics. For example, this framework can be adapted to constant-potential simulations. While AIMD may become computationally demanding for increasingly complex electrochemical environments, the emergence of machine learning interatomic potentials can offer a promising avenue to extend this framework to larger systems and longer time scales while maintaining near first-principles accuracy. The framework is transferrable to interfacial PCET in the context of multistep electrocatalysis and enables further molecular-level understanding of electrochemical reaction kinetics.

## Supplementary Material



## Data Availability

The AIMD trajectories, geometries used for vertical energy gap sampling, and input files are available in the Zenodo data repository at https://zenodo.org/records/22147013.

## Notes

Additional computational methods, AIMD trajectory analysis, vertical energy gap analysis, and sample input files (PDF)
