# Supplementary material for: Electrochemical Proton-Coupled Electron Transfer Free Energy Surfaces from Ab Initio Molecular Dynamics
Source: J Phys Chem Lett. 2026 Oct 26;17(44):12729–35. doi: 10.1021/acs.jpclett.6c02996 (PMC13746805; doi:10.1021/acs.jpclett.6c02996)
Supplement: Supplementary file 1 [file jz6c02996_si_001.pdf]

Supporting Information

Electrochemical Proton-Coupled Electron Transfer Free Energy Surfaces from *Ab Initio* Molecular Dynamics

Mahmudul Hasan, Robert E. Warburton*

Email: rew134@case.edu

Department of Chemical and Biomolecular Engineering
Case Western Reserve University
Cleveland, Ohio, 44106

SI Contents

S1. COMPUTATIONAL METHODS 2
S1.1. DFT geometry optimizations and single point calculations 2
S1.2. AIMD simulations 2
S1.2.1. Berendsen thermostat 2
S1.2.2. Nosé–Hoover thermostat 3
S1.2.3. Langevin dynamics 3
S1.3. Water/Pt(111) interface model 3
S2. ANALYSIS OF AIMD TRAJECTORIES 4
S2.1. RDF and water density profile 4
S2.2. VDOS 4
S2.3. DOS and kinetic energy partitioning 5
S3. TEMPERATURE PROFILES ALONG THE TRAJECTORIES 5
S4. SOLVATION STRUCTURE 6
S5. VERTICAL ENERGY GAP CALCULATION FROM THE DIABATIC STATES 13
S5.1. Structure generation 14
S5.1.1. Reactant and vertical product state structures from the reactant AIMD trajectory 14
S5.1.2. Product and vertical reactant structures from the product AIMD trajectory 14
S5.2. Vertical energy gap calculations 15
S6. PROBABILITY DISTRIBUTION FUNCTIONS AND FREE ENERGY SURFACES 16
S7. SAMPLE INPUT FILES 19
S7.1. Optimized and starting structures to initiate AIMD 19
S7.1.1. Reactant 19
S7.1.2. Product 22
S7.2. Geometry optimization input file with ASE 26
S7.3. Single point calculation input file 27
S7.4. AIMD input file (Berendsen dynamics in Quantum ESPRESSO) 29
S7.5. AIMD input file (Nosé–Hoover in Quantum ESPRESSO) 30
S7.6. AIMD input file (Langevin dynamics with ASE) 32
REFERENCES 35

S1. Computational methods

S1.1. DFT geometry optimizations and single point calculations

All density functional theory (DFT) calculations were carried out using the Quantum ESPRESSO (QE)[1,2] package within Atomic Simulation Environment (ASE).[3] The electronic structure was described within the generalized gradient approximation of Perdew, Burke, and Erzerhof (PBE) with D3 dispersion corrections.[4,5] We employed ultrasoft pseudopotentials to model the core electronic states[6] and the valence states were modeled using a plane-wave basis set with kinetic energy cutoffs of 40 Ry for wavefunctions and 410 Ry for charge density. The Brillouin zone was sampled with a 2×2×1 k-point grid and electronic occupations were treated using Gaussian smearing with a width of 0.01 Ry. The self-consistent field (SCF) convergence threshold was set to 10^{-6} Ry, with mixing parameters of 0.1 and a mixing dimension of 12 to ensure stable convergence.

Geometry optimizations were performed using the BFGS algorithm with ASE.[3] Structures were optimized until the maximum force on each atom was below 0.05 eV/Å. Single-point calculations and hydrogen-only partial geometry optimizations for vertical energy gap sampling were carried out using the same computational setup. For these calculations, the grand-canonical SCF (GCSCF) methods was employed to calculate energy gaps at constant potential.[7] These calculations specified the average of the Fermi energy from the *ab initio* molecular dynamics (AIMD) trajectories of reactant state as the target Fermi energy in the GCSCF. Note that the purpose of the GCSCF calculation herein is to account for charging contributions to the total energy between reactant and product states, and that other analytical and extrapolation methods could be used to account for these energy corrections.[8–13] Sample of the input files and coordinates of the atoms can be found in the **Section S7**.

S1.2. AIMD simulations

All AIMD simulations were performed using QE with PBE-D3. Brillouin zone sampling was restricted to the Γ-point, and SCF convergence was set to 10^{-4} - 10^{-7} Ry with a mixing parameter of 0.1 and mixing dimension of 12. The Pt-water interface was modeled using the effective screening medium (ESM) approach with esm_bc = bc3 and esm_w = 0.1. In case of Berendsen and Nosé–Hoover, ion motion was propagated using Newton's equations via the Verlet algorithm. To improve numerical stability, hydrogen atoms were assigned the mass of deuterium (2 amu) and temperature is set to 350 K, enabling stable integration at the time step of approximately ~1.0 fs (20.67 a.u.).

S1.2.1. Berendsen thermostat

AIMD simulations using the Berendsen thermostat[14] were performed within QE with SCF convergence of 10^{-4} Ry.

S1.2.2. Nosé–Hoover thermostat

AIMD simulations employing the Nosé–Hoover thermostat[15] were also performed using the built-in implementation in QE to ensure proper canonical ensemble sampling. SCF convergence thresholds were tried in the range of 10^{-4}, 10^{-5}, 10^{-6}, and 10^{-7} Ry. Temperature was maintained at 350 K using a Nosé–Hoover chain thermostat with three chains and a thermostat frequency parameter of 20 THz.

S1.2.3. Langevin dynamics

Langevin dynamics[16] simulations were carried out using QE coupled with ASE, where the Langevin dynamics is implemented at the ASE. The temperature was maintained at 350 K using a friction coefficient of 0.01 fs^{-1}. This combined QE–ASE framework enables efficient implementation of stochastic dynamics not natively available in QE.

S1.3. Water/Pt(111) interface model

The Pt–water interface was modeled using a three-layer 3×3 Pt(111) slab from an optimized bulk lattice constant of 3.9431 Å. Explicit water molecules were included with cell parameters fixed at a = 8.3644 Å, b = 7.2438 Å, c = 60 Å. Partial constraints applied to mimic bulk-like behavior of the slab by fixing bottom two layers of Pt(111) while relaxing surface and solvent degrees of freedom. The top of the slab was kept at z=0 in the unit cell to apply ESM boundary condition.[17] The slab was initially solvated using the TIP5P water[18] with 31 molecules of water in GROMACS,[19] which corresponds to a ~15 Å water slab at the Pt(111) surface. The reactant state model for AIMD was initialized using bare Pt(111) slab solvated by 31 water molecules. The product state model introduced one additional hydrogen atom, which was adsorbed on the FCC site of the Pt(111) surface while keeping all the water molecules intact. We note that the stoichiometry is different between calculations derived from the reactant and product AIMD trajectories. In turn, the vertical state for the product trajectory corresponds to the quantized proton localized to a solvated H_2O molecule, representing a H_3O^+ ion (see top-right in **Figure 1** and **Figure S13D**). It is challenging to maintain the stoichiometry while retaining the definitions of the diabatic states between the two trajectories because otherwise the ions are not charge balanced and do not remain solvated, either dissociating or adsorbing to the metal surface. We note that the stoichiometry is maintained when comparing energetics in ΔE calculations, and that reorganization energies account for the energetics of both outer-sphere solvent and inner-sphere proton and surface–adsorbate reorganization. Future studies could adapt the methods presented herein to include the effects of countercharges and/or counterions in the simulation cell.

The system was subject to an initial geometry optimization in QE prior to equilibration with AIMD. We used the effective screening medium (ESM) vacuum/slab/metal (bc3) boundary condition to screen interactions across the periodic boundary normal to the slab and to enable GCSCF calculations. This approach includes applies a planar slab of a fictitious perfect conductor

(ESM-metal) to screen charges and dipoles at *z*/2 position and a vacuum (ESM-vacuum) at -*z*/2 position of the unit cell (**Figure S1**).[17] The length of the unit cell along the normal of the Pt(111) surface was 60 Å, which allowed ~15 Å of vacuum between the top layer of the water and ESM-metal and 30 Å of vacuum between the bottom layer of Pt(111) and ESM-vacuum, which screens the net charge in the cell. Periodic boundary conditions had been maintained along other directions.

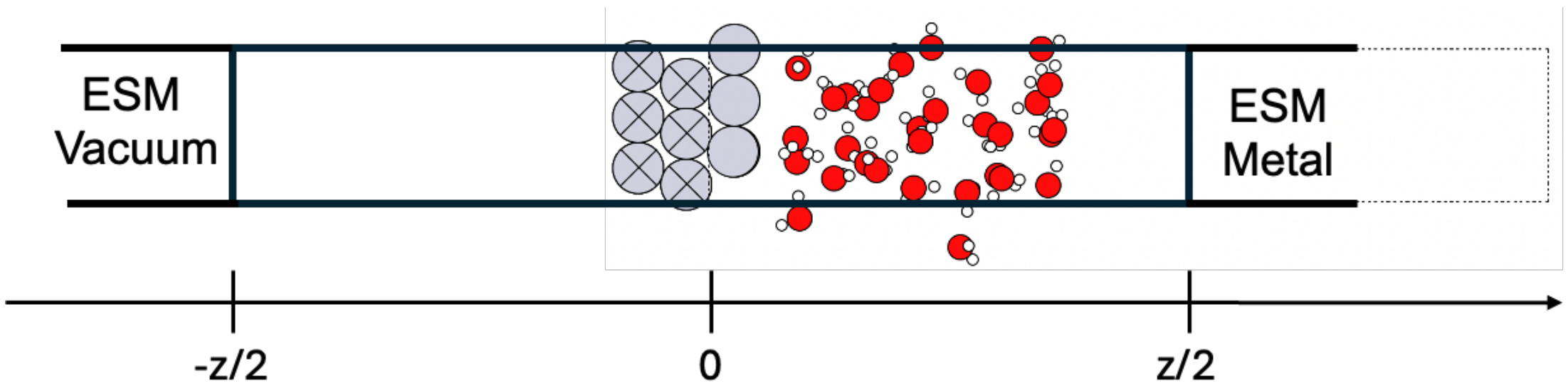

Figure S1. Model of water/Pt(111) interface with a vacuum/slab/metal (bc3) ESM boundary condition.

S2. Analysis of AIMD trajectories

S2.1. RDF and water density profile

The local structure of interfacial water was characterized through the calculation of radial distribution functions (RDFs) using the TRAVIS package.[20] RDFs for the O–O, O–H, and H–H atomic pairs were first evaluated to investigate the short-range ordering and hydrogen-bonding structure of the water network at the interface. A maximum correlation distance of 20 Å and 600 histogram bins were employed, and the RDF was normalized using the radial distribution correction implemented in TRAVIS.

The water density profile normal to the Pt(111) surface was calculated from the oxygen atom distribution obtained from equilibrated AIMD trajectories. Oxygen atoms were binned along the surface-normal direction with a bin width of 0.1 Å, and the resulting histogram was normalized by the number of frames, the average surface area of the simulation cell, and the bin thickness to obtain the oxygen number density profile. Assuming a one-to-one correspondence between oxygen atoms and water molecules, the density was subsequently converted from $Å^{-3}$ to g cm^{-3}, which enables a detailed characterization of water layering and structural ordering at the water/Pt(111) interface (**Figure S1**).

S2.2. VDOS

The vibrational density of states (VDOS) of the Pt–water interface was calculated using the TRAVIS analysis package.[20] The vibrational spectrum was obtained from the Fourier transform of the mass-weighted velocity autocorrelation function (VACF) using the power spectrum analysis implemented in TRAVIS.[21] Molecule recognition was performed while excluding Pt atoms from bond recognition to ensure robust identification of water molecules. The analysis used a trajectory

sampling interval of 1 fs. 10,000 equilibrated trajectory frames were analyzed with an autocorrelation depth of 2000 frames with mass weighting.

S2.3. DOS and kinetic energy partitioning

Density of states (DOS) and kinetic energy partitioning were performed using the DoSPT package[22] within the two-phase thermodynamic (2PT)[23] framework. The equilibrated 10 ps molecular dynamics trajectories were analyzed. AIMD trajectories were fed to DoSPT in XYZ format, and atomic velocities were reconstructed from atomic coordinates using the velocity estimation procedure implemented in DoSPT. For kinetic-energy partitioning in **Figure S10**, each water molecule was defined as an individual subgroup containing one oxygen and two hydrogen atoms, whereas each Pt atom was treated as a separate subgroup. Gaussian smoothing was applied to the density-of-states spectra with a broadening parameter 0.3 ps^{-1}. The vibrational density of states (VDOS), rotational density of states (RDOS), and translational density of states (TDOS) allow for the characterization of vibrational, rotational, and translational motions of species at the Pt–water interface. These decomposed spectra were subsequently used to partition the kinetic energy among the different modes of motion and to evaluate thermodynamic properties within the 2PT formalism.

S3. Temperature profiles along the trajectories

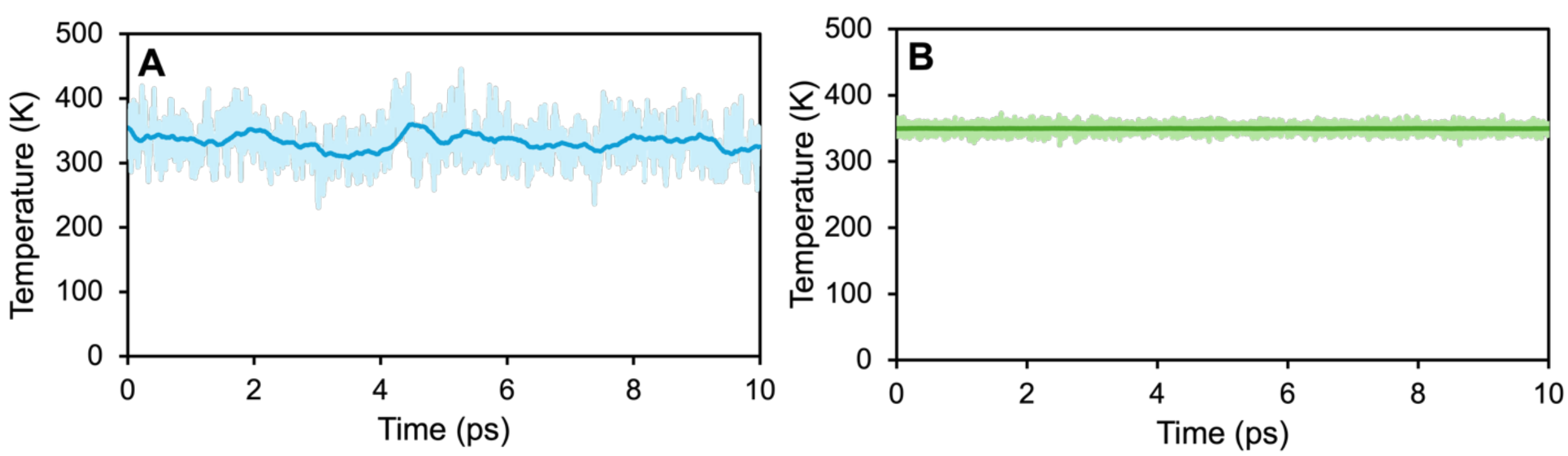

Figure S2. Equilibrated temperature profile of the reactant state with (**A**) Langevin dynamics and (**B**) Berendsen thermostats, each at an SCF convergence threshold of 10^{-4} Ry. Darker curves correspond to 500 fs moving average.

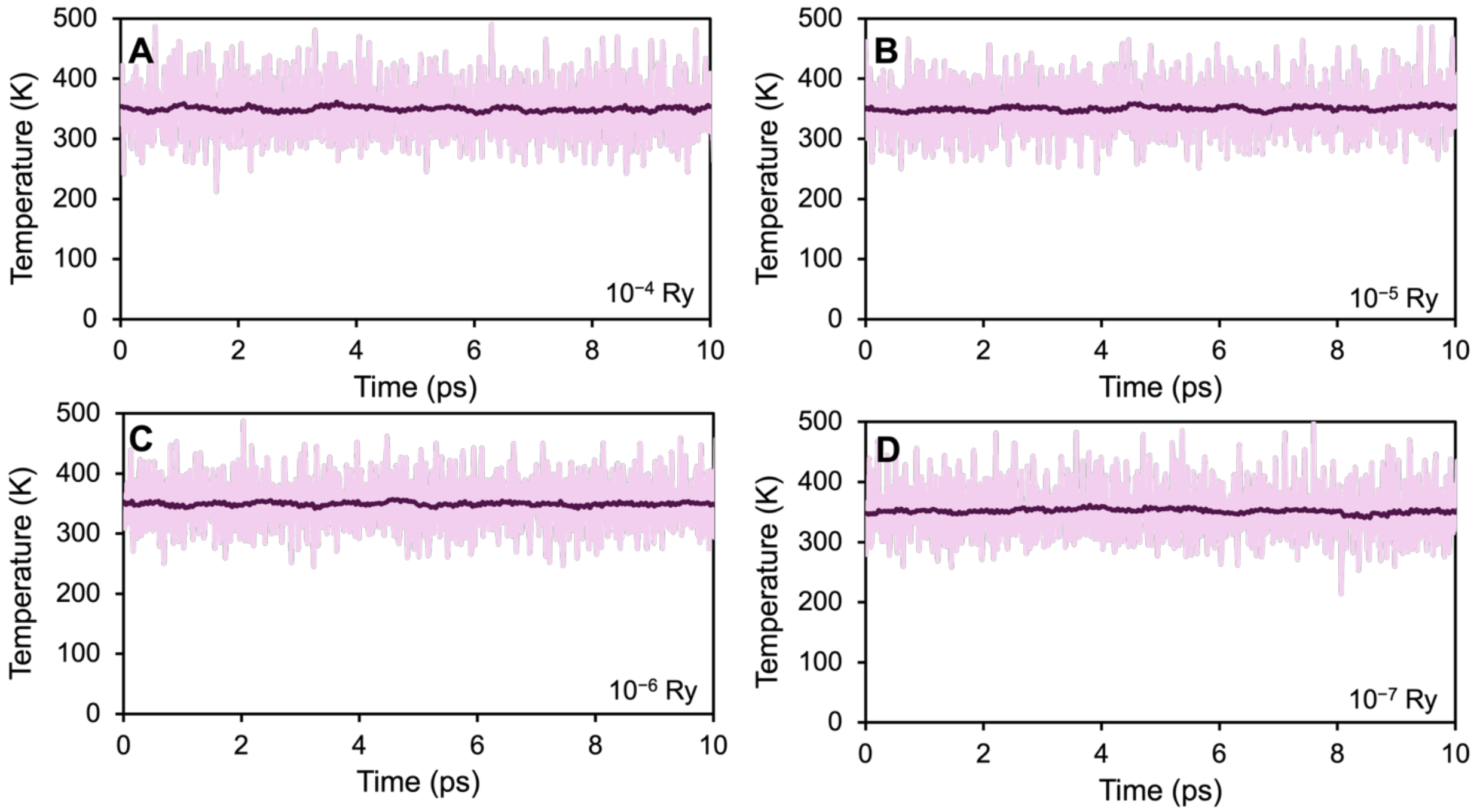

Figure S3. Equilibrated temperature profile of the reactant state with Nosé–Hoover thermostat at SCF convergence thresholds of (**A**) 10^{-4}, (**B**) 10^{-5}, (**C**) 10^{-6}, and (**D**) 10^{-7} Ry. Darker curves correspond to 500 fs moving average.

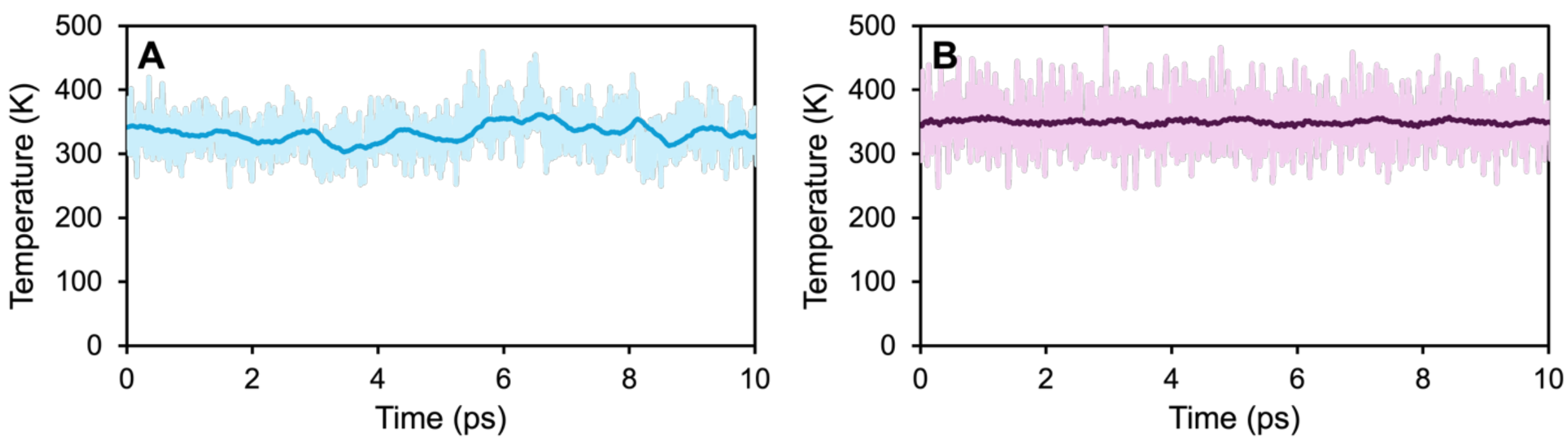

Figure S4. Equilibrated temperature profile of the product state with (**A**) Langevin dynamics at convergence threshold of 10^{-4} Ry and (**B**) Nosé–Hoover thermostat at convergence threshold of 10^{-7} Ry. Darker curves correspond to 500 fs moving average.

S4. Solvation structure

The solvation structure of the interface is analyzed by calculating the radial distribution functions (RDF) and coordination number of atomic pairs in **Figure S5** and water density profile in **Figure S6** for the 10 ps production run of the equilibrated AIMD trajectories. The O–H RDF (**Figure S5A**) exhibits a first peak at 0.95 Å associated with the covalent bond between the H and O in water,

where the corresponding coordination number of two represents that two H atoms covalently bonded with the O atoms. The second peak corresponds to the H atom of water in first solvation shell, which forms H-bonds to the surrounding O atoms of other water molecules. The coordination number at 2.55 Å is 3.9 ~ 4, which originates from the two covalent bonds of O with H and two H-bond with surrounding water molecules. The correlation between H-bonded neighbors is also contained in the 1st peak of the O-O RDF (**Figure S5B**). The sharp and intense peak at 2.75 Å of O-O RDF indicates the strong and H-bonded hydration shell. At 4.15 Å, a broad 2nd peak appeared represents the second coordination shell, where water is more loosely bound and disordered. The first peak at 1.55 Å of H-H RDF (**Figure S5C**), is attributed to the H atoms belonging to the same water molecule and the peak at 2.25 Å represents the H atoms of the nearest neighboring water molecules, which bonded by H-bonding. The RDFs between the different thermostats show similar trends, although the Berendsen thermostat slightly underestimates the nearest-neighbor H_2O peak in the O–H and O–O RDFs in comparison to the Langevin and Nose-Hoover. Overall, the RDF analysis shown in **Figure S5** represents the structure of the water molecules at the interface, in agreement with the previously reported results.[24–26]

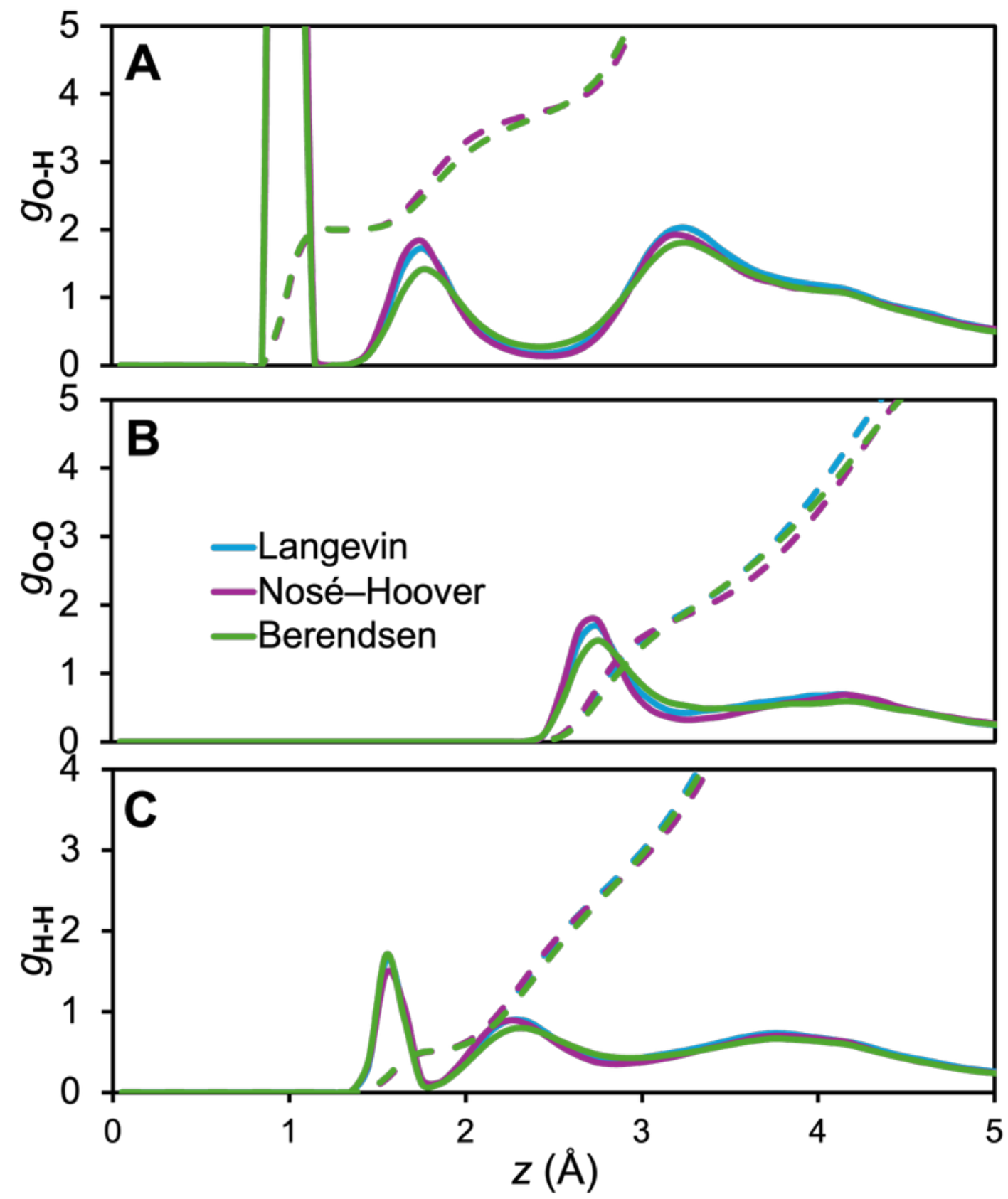

Figure S5. Radial distribution functions (*g*, solid) and cumulative coordination numbers (dashed) of (**A**) O-H, (**B**) O-O, and (**C**) H-H pairs at different thermostats at convergence threshold of 10^{-4} Ry. Note that the coordination number is obtained from integration of the radial distribution function.

The interfacial water density profile (**Figure S6**) is calculated by averaging the oxygen distribution with respect to the surface of the slab from the equilibrated trajectories. Two sharp peaks at 2.15 and 3.05 Å from the Pt surface represents the formation of the bilayers of the interfacial water molecules, which is in excellent agreement with the previously reported results.[27,28] The area of the first and second peaks are not same, which is inconsistent with the formation of ice-like hexagonal bilayer structure, where one half of water molecule is arranged flatly on the surface and another half in H-up or H-down configuration. This discrepancy arises from the dynamic behavior of water molecule at the interface. Sakong and Groß previously showed that the waters represented by the first peak are bonded to Pt surface through O and arranged flatly, while the second peak corresponds to the H-up or H-down,[29] which is in agreement with our findings. A representative snapshot from the final frame of the Langevin AIMD trajectory is shown in **Figure S6**. Water molecules from the first peak region frequently exchange with the second peak waters and water from the bulk region is also frequently exchanged with the second peak waters, which causes the rise of second peak.[30] After the second peak, which ends at 4.44 Å, the water molecules are distributed uniformly and maintain the bulk water density of ~1 g cm^{-3}.

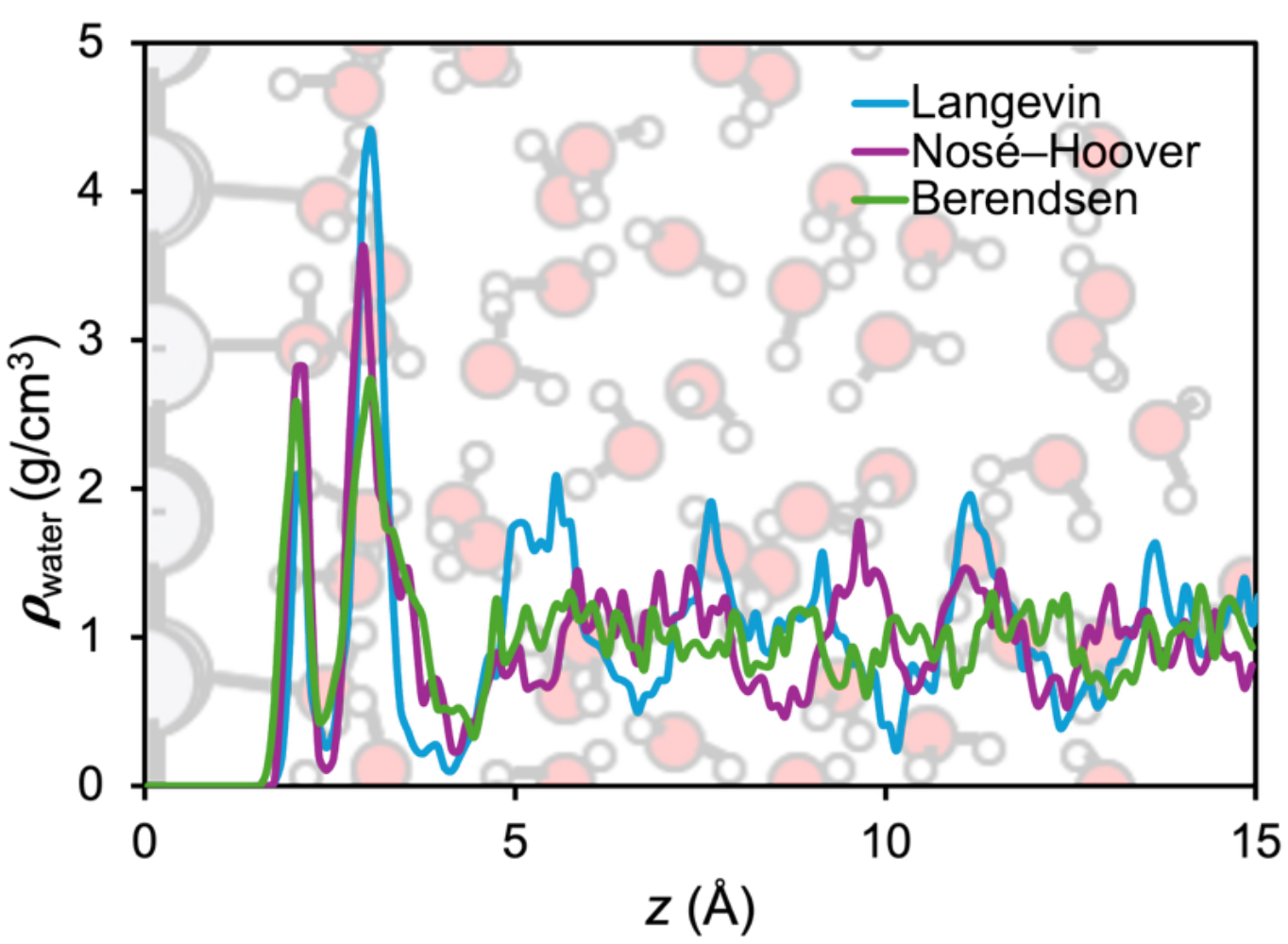

Figure S6. Density profile of water with respect to the surface of the Pt(111) of the reactant state at different thermostats with convergence threshold of 10^{-4} Ry.

Vibrational density of states (VDOS) of the Pt/water interface is shown in **Figure S7**. As we used heavy deuterated water (D_2O) the VDOS peaks are red-shifted by an order of approximately $\sqrt{2}$ compared to the H_2O. The peaks around 2500, 1200, 400, and 70 cm^{-1} represent OH stretching, bending, liberational mode and translational H-bond stretching respectively.[31,32] Here, the effect of thermostats is compared for the same system. Other than the peak intensity, the peak positions of the different modes are similar across the three thermostats considered. The water density of profile in **Figure S6**, also shows that the decrease of the peaks for Berendsen, which

proves the presence of less water molecules in the interface compared to other two thermostats and confirms the weak interaction between Pt and water.

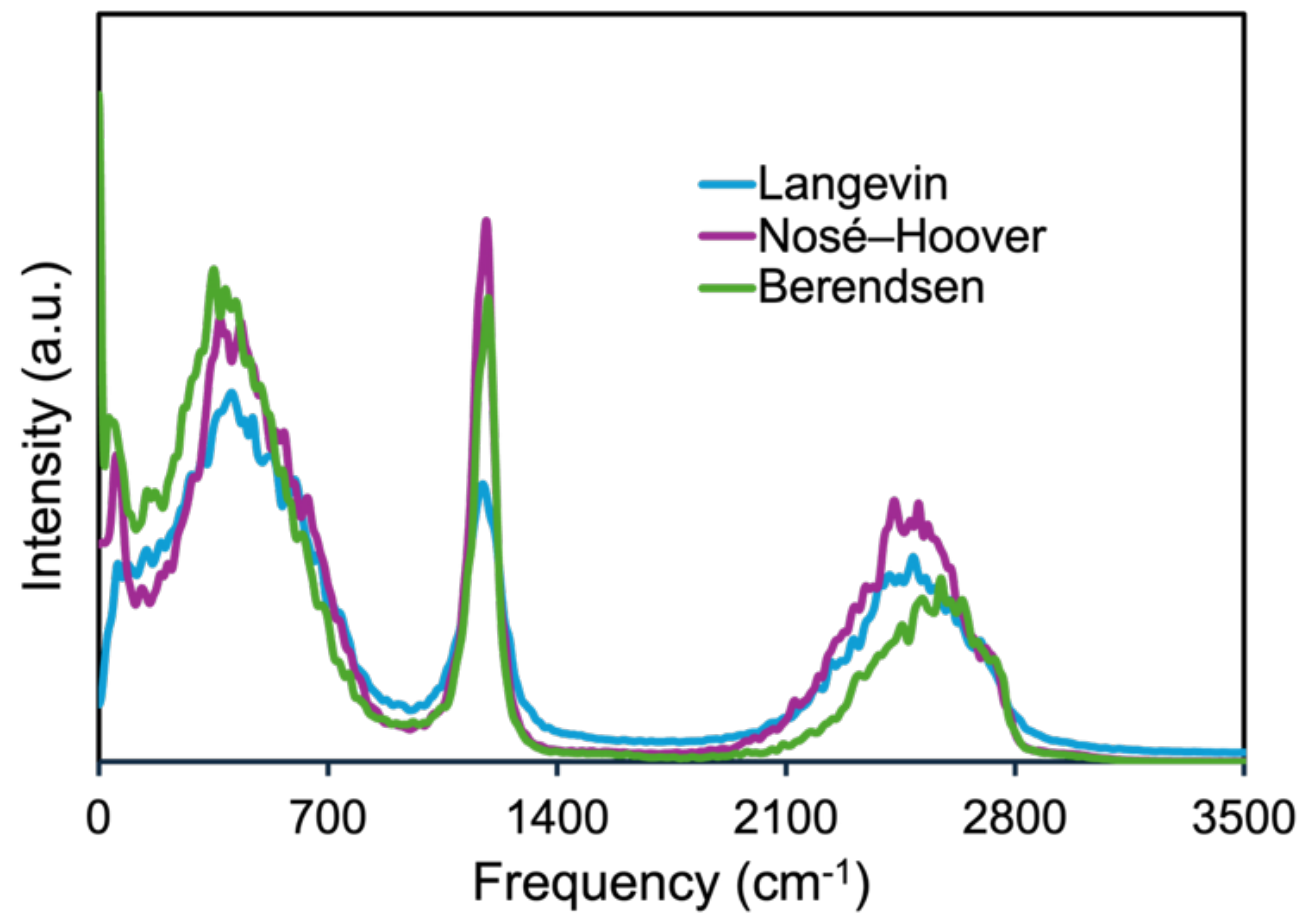

Figure S7. Vibrational density of state of the reactant state at different thermostats with convergence threshold of 10^{-4} Ry.

It is also observed that the peak intensity for the Berendsen at the higher frequency is lowest and at lower frequency is the highest, which is in part suggestive of the "flying ice cube effect". Flying ice cube effect can be defined as an artifact in which the kinetic energy of high frequency vibrational modes are drained into low frequency translational and rotational modes.[33] To further assess this artifact, density of states (DOS) for water has been calculated and compared in **Figure S8** for the reactant state with different thermostats at the convergence threshold of 10^{-4} Ry. The number of degrees of freedom (DOF) can be found by integrating the peak areas, and the theoretical total DOF of water is $3N$, where N is the total number of water molecule;[33] these results are summarized in **Table S1**. It is observed from the **Figure S8** and **Table S1** that Berendsen thermostat shows significantly lower VDOS and higher RDOS and TDOS. Moreover, kinetic energy contribution from VDOS is significantly lower and from RDOS is significantly higher compared to the theoretical trends (**Figure S10A**) which confirms the flying ice cube effect. Nosé–Hoover with 10^{-4} Ry shows the opposite incident (**Figure S10B**) i.e. transfer of kinetic energy from lower frequency to higher frequency mode, confirms the reverse flying ice cube effect. Previous work suggests that it is possible to avoid these artifacts with Nosé–Hoover thermostat by tightening SCF convergence criteria.[33] The DOS calculated from Nosé–Hoover trajectories with different SCF convergence thresholds is compared in **Figure S9** and DOF is summarized in **Table S2**. These data show that Nosé–Hoover thermostat with convergence threshold of 10^{-7} Ry has DOF closer to the theoretical values and kinetic energy contribution from each of the DOS almost coincides with the theoretical trends (**Figure S10C**). Langevin dynamics with 10^{-4} Ry shows the similar results (**Figure S10D**), which confirmed that Nosé–Hoover with 10^{-7} Ry and Langevin dynamics with 10^{-4} Ry successfully circumvent the artifact of flying ice cube effect and provide practical trajectories for kinetic analysis. AIMD trajectories for the product state have been generated using the optimized thermostats and convergence criteria (**Figure S4**). Though Langevin

dynamics with looser convergence criteria generate good quality of trajectory, these dynamics were performed using ASE because Langevin dynamics are currently not directly available as a built-in thermostat in the QE. On the other hand, Nosé–Hoover is available in recent QE releases, which results in significantly faster simulations through bypassing the ASE conduit for molecular dynamics, even at tighter convergence criteria.

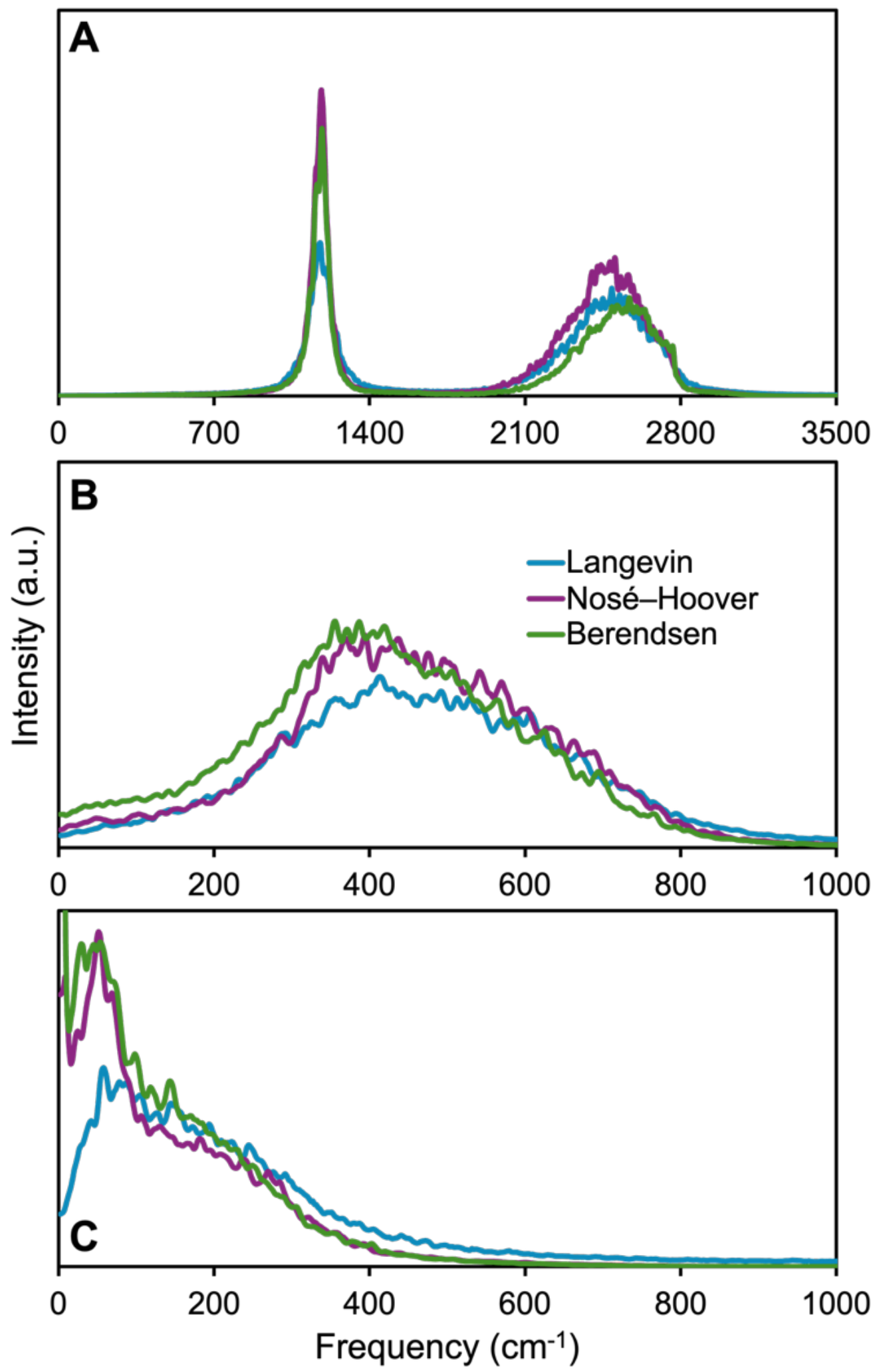

Figure S8. (**A**) Vibrational density of states (VDOS), (**B**) rotational density of states (RDOS), and (**C**) translational density of state (TDOS) of water of the reactant state at different thermostats with an SCF convergence threshold of 10^{-4} Ry.

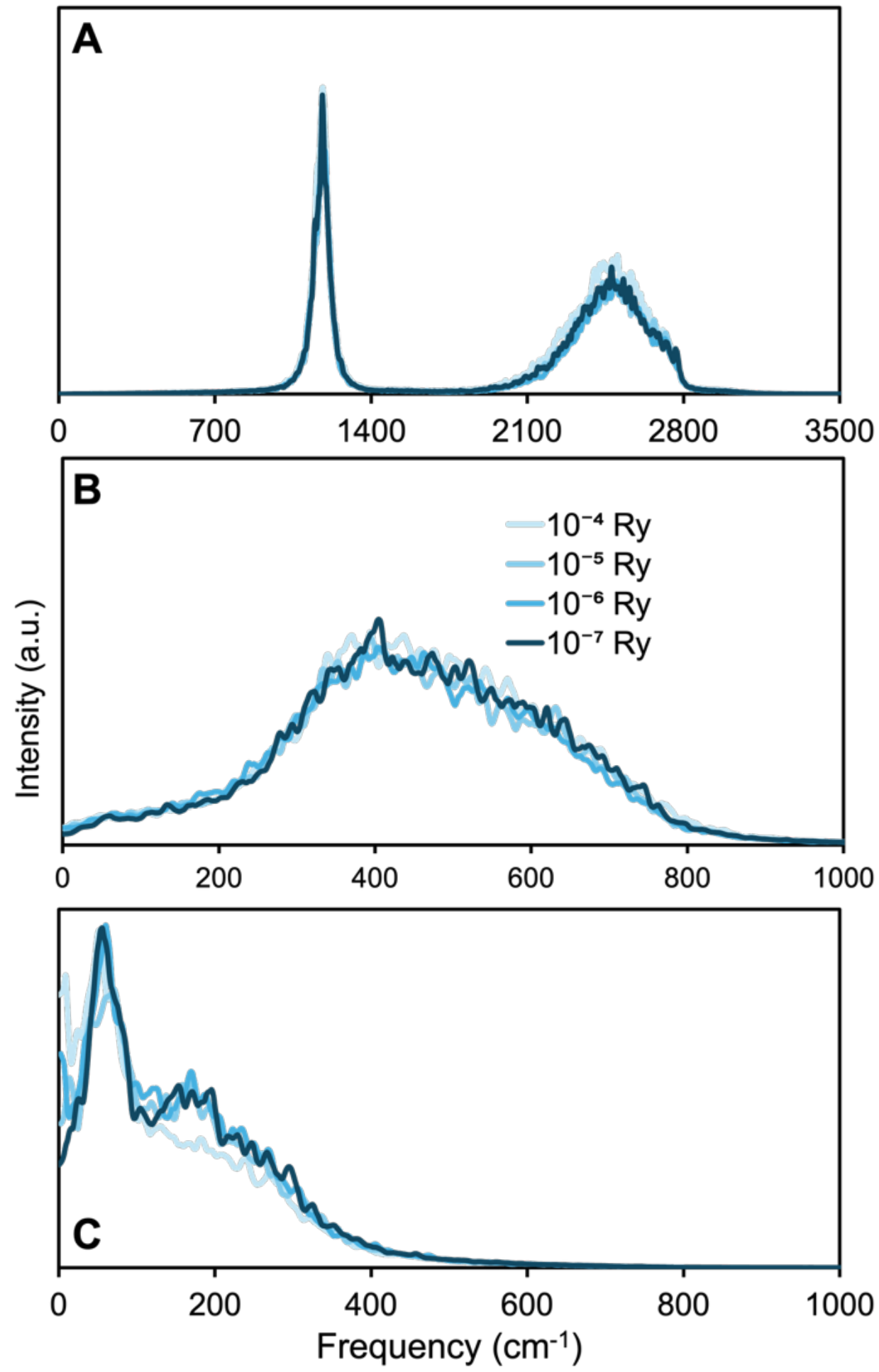

Figure S9. (**A**) VDOS, (**B**) RDOS, and (**C**) TDOS of water of the reactant state with Nosé–Hoover thermostat with convergence threshold of 10^{-4}, 10^{-5}, 10^{-6}, and 10^{-7} Ry.

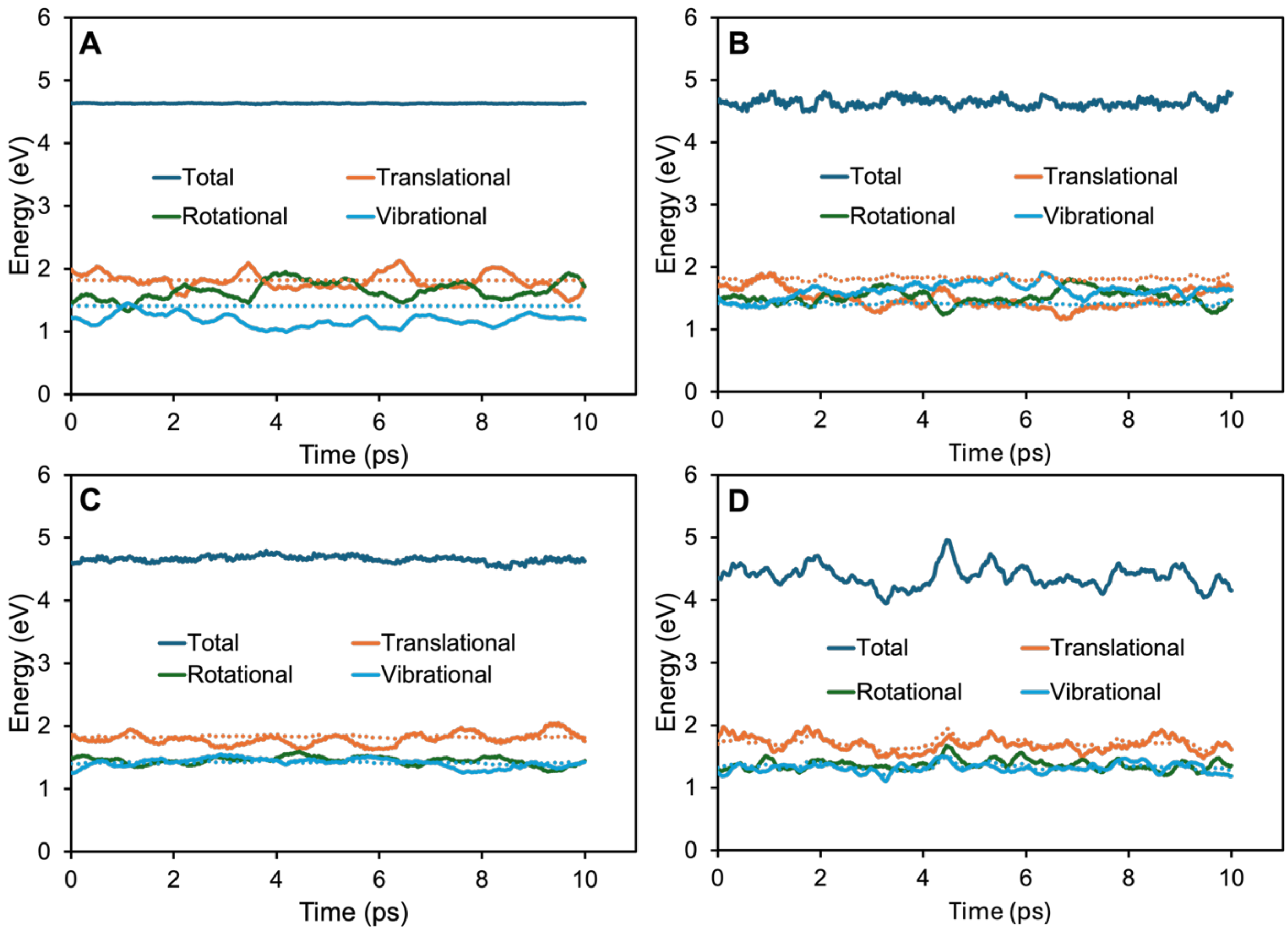

Figure S10. Contribution of the vibrational, rotational, and translational kinetic energy to the total kinetic energy in reactant state with (**A**) Berendsen thermostat at 10^{-4} Ry, (**B**) Nosé–Hoover thermostat at 10^{-4} Ry, (**C**) Nosé–Hoover thermostat at 10^{-7} Ry, and (**D**) Langevin dynamics at 10^{-4} Ry.

Table S1. Degrees of freedom (DOF) of water of the reactant state with different thermostats at convergence threshold of 10^{-4} Ry.

DOS	Theoretical	Langevin	Nosé–Hoover	Berendsen
VDOS	93	87	105	79
RDOS	93	92	100	105
TDOS	93	94	89	105
Total	279	273	294	289

Table S2. DOF of water of the reactant state with Nosé–Hoover thermostat with convergence threshold of 10^{-4}, 10^{-5}, 10^{-6}, and 10^{-7} Ry.

**DOF**	**Theoretical**	10^{-4} Ry	10^{-5} Ry	10^{-6} Ry	10^{-7} Ry
VDOS	93	105	85	83	89
RDOS	93	100	93	93	96
TDOS	93	89	92	98	94
Total	279	294	271	274	279

In **Figure S11**, we compared the solvation structure of water at the interface. As both of the states are simulated at zero total charge, the water at interface has a similar geometric and vibrational structure in each state.

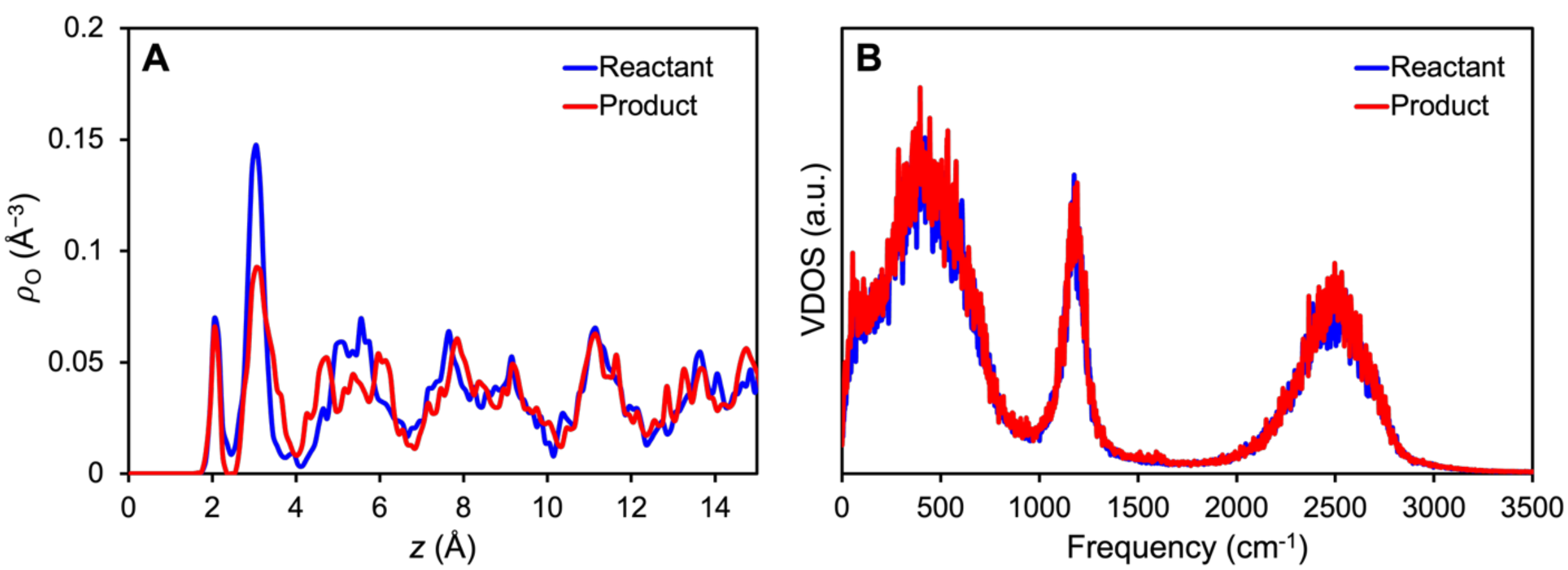

Figure S11. Comparison of A) oxygen density profile and B) VDOS of reactant and product states.

S5. Vertical energy gap calculation from the diabatic states

To determine the position of the proton donating water molecule, we performed calculations for several possible proton donating water molecules located at different distances from the interface. As shown in **Figure S12**, the ΔE has similar values for all the proton donating bulk water molecules, selection of transferring proton from any of these water molecules expected to yield a similar ΔE distribution. In this study, we selected the proton donating water molecule at ~ 10 Å from the surface of Pt(111) and center of the unit cell, which is in the bulk region. The transferring protons involved in proton-coupled redox reactions at electrode surfaces are conducted through the interfacial hydrogen-bonding network of water via the Grotthuss mechanism.[34] We note that protons exhibit nuclear quantum effects along the water chains that constitute the Grotthuss proton

relay between the interface and bulk electrolyte.[35–38] Given these effects and because the net PCET reaction occurs when an electron is transferred to or from the external circuit and an ion (e.g., proton, hydroxide, or other ion) enters or leaves the electric double layer to maintain charge neutrality, we chose the proton donor and acceptor water molecules from the bulk region. Moreover, keeping proton far from the surface also allows us to have stable simulations that maintain the definitions of the two diabatic states of the PCET reaction.

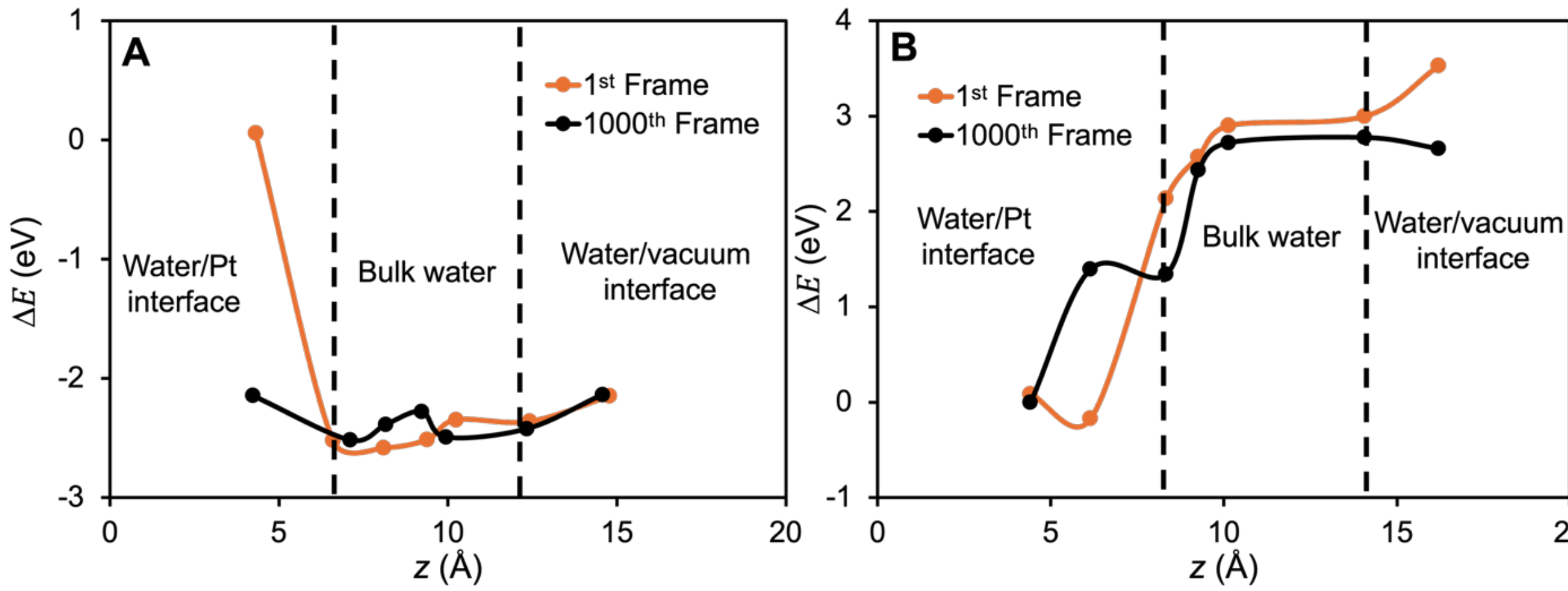

Figure S12. Comparison of vertical energy gap for 1st and 1000th frames obtained from the A) reactant state and B) product state as a function of the distance of the proton donating water molecule from the Pt(111) surface.

S5.1. Structure generation

S5.1.1. Reactant and vertical product state structures from the reactant AIMD trajectory

Snapshots were taken from the equilibrated reactant trajectories at an interval of 40 fs. For each selected snapshot, the original AIMD configuration was retained as the reactant state as shown by the representative example in **Figure S13A**. The corresponding vertical product state was generated using an automated proton placement procedure implemented in ASE. In this procedure, a H atom from a selected water molecule is placed at a predefined coordinate associated with adsorption to an fcc site on Pt(111), as shown in **Figure S13B**. Note that the transferring quantized proton is shown in cyan in each panel of **Figure S13**. This procedure was repeated for all the selected snapshots along the trajectory, which generates 250 sets of reactant and vertical product state structures for vertical energy gap sampling.

S5.1.2. Product and vertical reactant structures from the product AIMD trajectory

Snapshots were taken from the equilibrated product trajectories at an interval of 40 fs, where the original AIMD configuration is retained as the product state at each snapshot (**Figure S13C**). As also described in Section S5.1.1, the corresponding vertical reactant state was generated using an automated proton placement procedure implemented in ASE. In this procedure, the adsorbed H on the Pt(111) surface was transferred to a predefined water molecule in a way that the distance

between the transferred H to the O of the water molecule is 1 Å and the mean distance from the transferred H to the H of the water molecule is 1.25 Å to avoid unphysical atomic overlap, as shown by the representative example in **Figure S13D.** This procedure was repeated for all the selected snapshots along the trajectory, which generates 250 sets of product and vertical reactant state structures for vertical energy gap sampling.

S5.2. Vertical energy gap calculations

We used the structure generation procedures described in **Sections S5.1.1** and **S5.1.2** to generate snapshots from the reactant and product AIMD trajectories for vertical energy gap calculations. Each snapshot corresponds to a set of nuclear coordinates $\mathbf{r}^N$, which includes all nuclei except the transferring quantized proton in its definition (shown in cyan in **Figure 13**). For the reactant state trajectory at a given snapshot's set of nuclear coordinates $\mathbf{r}^N$, the vertical energy gaps are calculated as the energy difference between: (1) a single point calculation of the reactant structure from AIMD (e.g., **Figure 13A**); (2) a partial geometry optimization of the quantized hydrogen adsorbed on the Pt surface (e.g., **Figure 13B**). Note that given the definition established by **Eq. 2** in the main text, the vertical energy gaps computed from the reactant trajectory are negative because the nuclei are in nonequilibrium configurations for the product state (c.f., main text **Figure 2A,C** and **Figure S15A,C**). Similarly, the vertical energy gaps calculated from the product trajectory are calculated as the energy difference between: (1) a single point calculation of the product structure from AIMD (e.g., **Figure 13C**); (2) a partial geometry optimization of the quantized hydrogen attached to a water molecule in the bulk of the solvent layer (e.g., **Figure 13D**). The energy gaps computed from the product trajectories are positive (**Figure 2B,D** and **Figure 15B,D**). As described in **Section S1.1**, the energy gap calculations were performed at constant potential using GCSCF DFT calculations to account for charging contributions in the energy difference calculations.

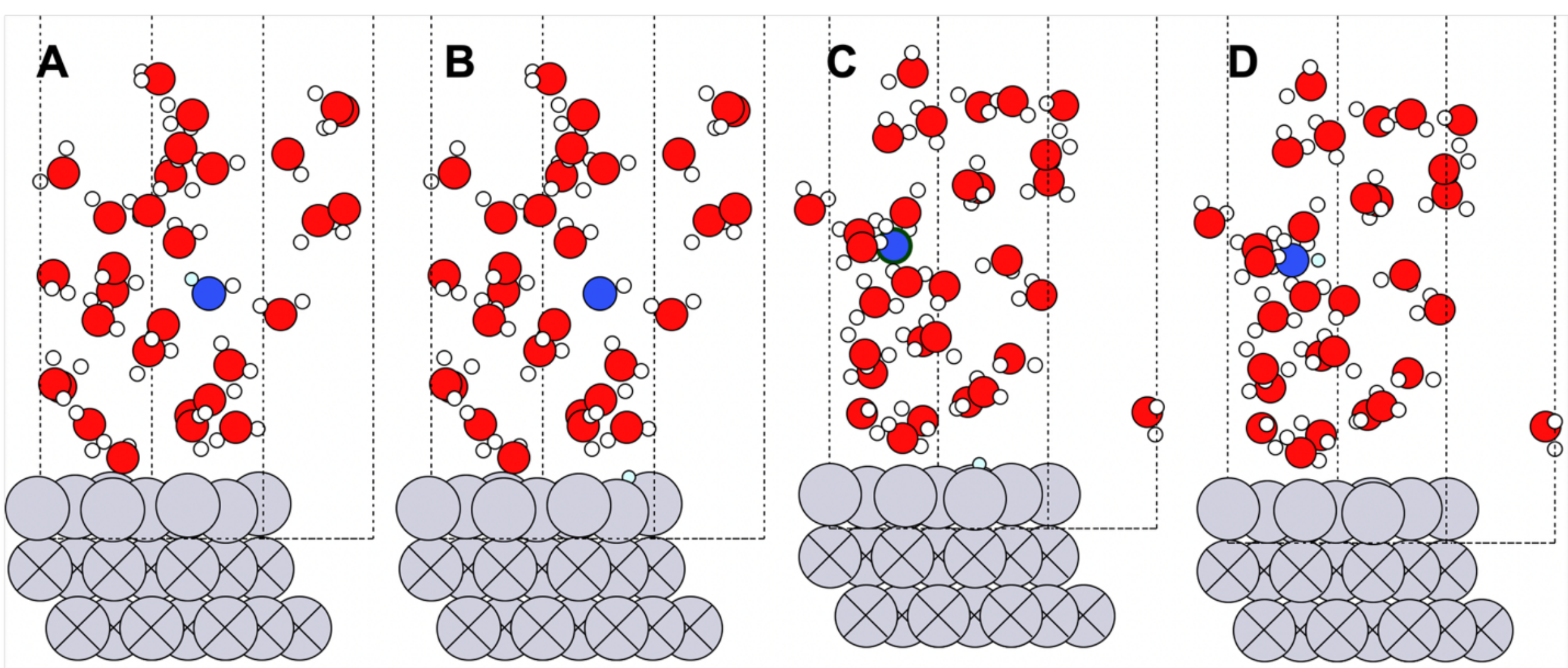

Figure S13. Schematic of representative snapshots used to calculate $\langle \Delta E \rangle_{\mathrm{R}}$ from the (**A**) 1st frame of the reactant state from the equilibrated reactant trajectory and (**B**) nonequilibrium localization of the quantized hydrogen to a top site on Pt(111). In (**A-B**),

the nuclear positions of all the atoms ($\mathbf{r}^N$) are same except the transferring quantized proton. (**C**) 1st frame of the product state from the equilibrated product trajectory, and (**D**) nonequilibrium localization of the quantized hydrogen to the bulk water molecule. In (**C-D**), the nuclear positions of all the atoms ($\mathbf{r}^N$) are same except the transferring quantized proton. These snapshots are taken from the trajectories generated by Langevin dynamics. Grey color represents Pt, red represents O, white represents H, blue represents the O proton donor, and cyan represents the transferring quantized proton.

S6. Probability distribution functions and free energy surfaces

The ΔE distributions calculated from snapshots along the reactant and product trajectories using Langevin and Nosé–Hoover thermostats are shown in **Figure 2C-D** and **Figure S15C-D**, respectively. The histogram was constructed using a bin width of 0.1 eV and normalized such that the total area under the histogram integrated to one. A Gaussian fit to the distributions was conducted to conform to the Marcus linear-response approximation. The fitting and Anderson-Darling test parameters are shown in **Table S3**. **Figure S14** shows that fitting parameters and reorganization energy are invariant beyond 5 ps, suggesting adequate statistical sampling. An analytical expression for the probability distribution function was obtained from the Gaussian fit. The probability distribution function was used to calculate the near-equilibrium free energies in each diabatic state using **Eq. 4**, which corresponds the lower points in **Figure 3** and **Figure S16**. Specifically, the energy gaps computed from the 250 AIMD snapshots for each trajectory are represented as explicit data points on the free energy curves, where the free energy of these points is determined from the probability distribution function using **Eq. 4.** The nonequilibrium energies were calculated using **Eq. 5**, where these values correspond to the higher-energy points in **Figure 3** and **Figure S16**. The parabolic fitting of the equilibrium and nonequilibrium energies yield the free energy surface of the respective states. To assess the goodness of the quadratic fit, we evaluated the residuals of the fits and obtained RMSE values of approximately 0.022 eV for both reactant and product free-energy surfaces. Although the parabolic fits reproduce more than 99.7% of the variance ($R^2 > 0.997$), we emphasize that R^2 is not a statistical test for the validity of the linear response approximation.

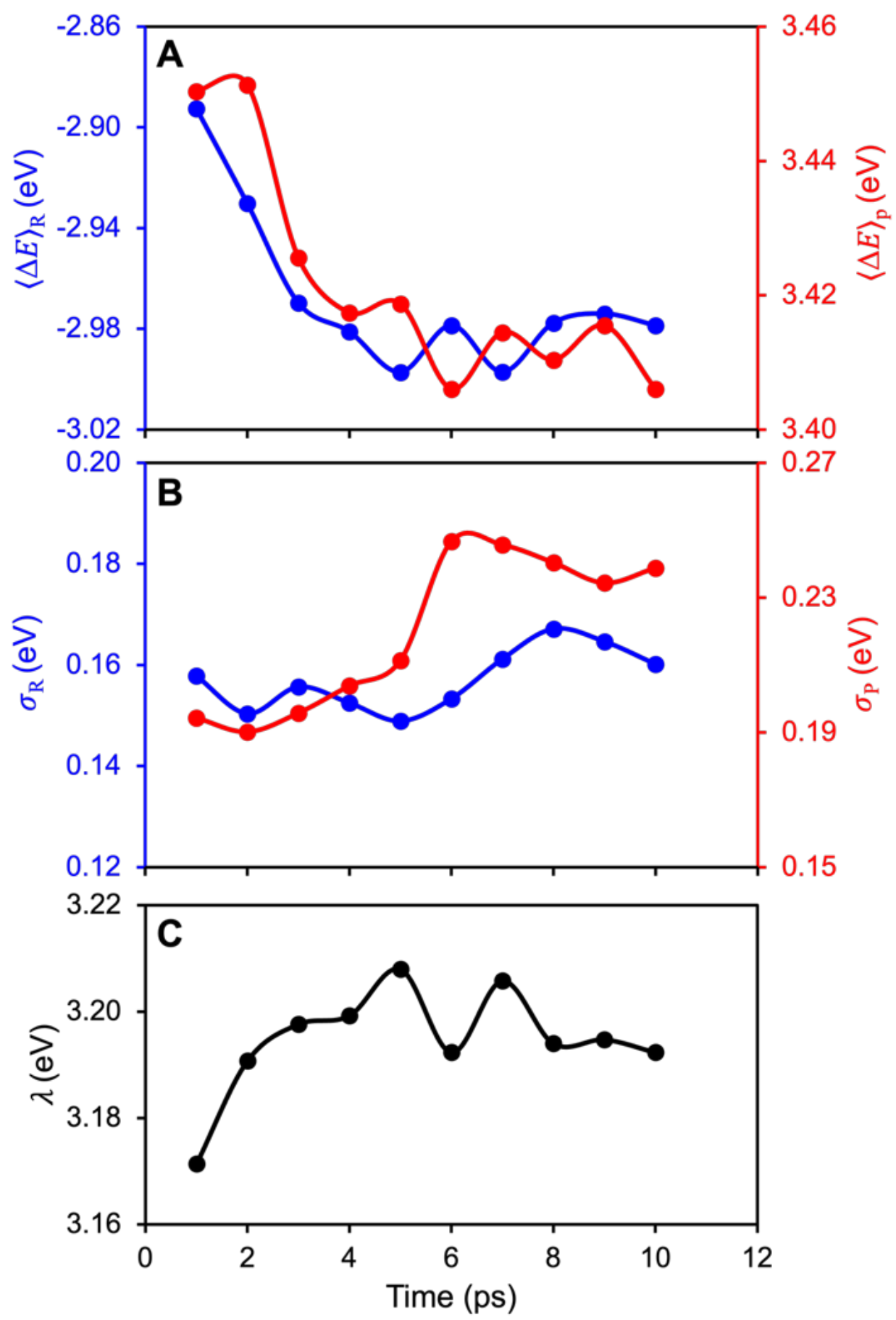

Figure S14. Variation of (**A**) $\langle \Delta E \rangle_M$ (**B**) σ_M, and (**C**) λ along the Langevin trajectories. These plots show the invariance of the parameters that describe the diabatic PCET free energy surfaces at 10 ps of the AIMD production run.

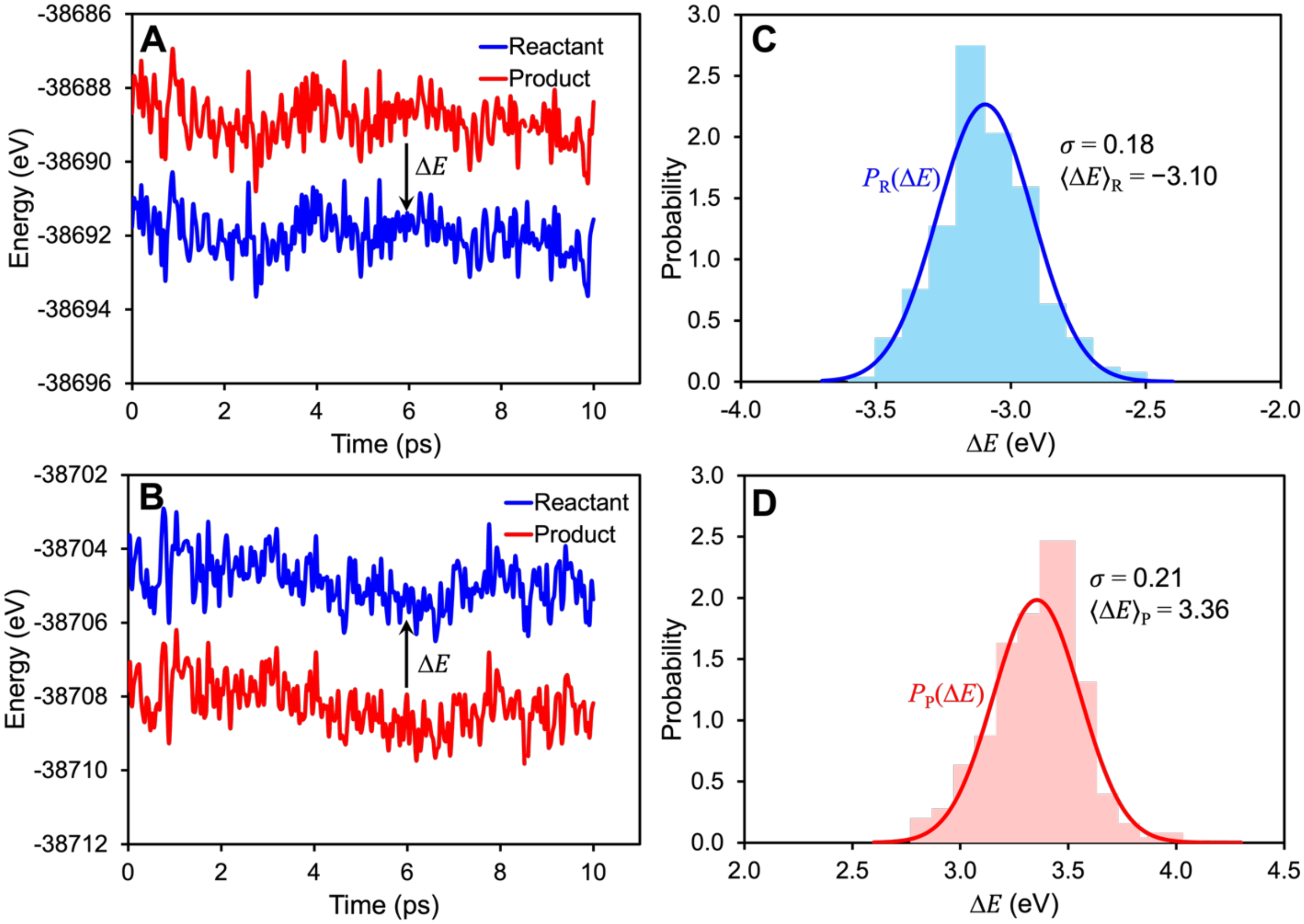

Figure S15. (**A-B**) Total energies along the AIMD trajectories of the (**A**) reactant and (**B**) product state. The low energy curves are obtained directly from the AIMD simulations and the high energy curves are computed based on the instantaneous vertical energy gap (ΔE) for the associated snapshot. (**C-D**) Calculated distribution of (ΔE) for (**C**) reactant and (**D**) product states. The probability distribution functions $P_M(\Delta E)$ are fit to a Gaussian distribution with mean $\langle \Delta E \rangle_M$ and standard deviation σ_M parameters included on the plots. Here, data is obtained from the trajectories with Nosé–Hoover thermostat with convergence threshold of 10^{-7} Ry. The histogram was constructed using a bin width of 0.1 eV and normalized such that the total area under the histogram is equal to 1.

Table S3. Gaussian fit parameters to ΔE probability distributions. A^2 is the Anderson–Darling test statistic for normality. The critical value threshold of A^2 for normality at standard significance level of α = 0.05 (5%) is 0.775.

Gaussian Fit Parameters	Reactant (Langevin)	Product (Langevin)	Reactant (Nosé–Hoover)	Product (Nosé–Hoover)
σ_M	0.16	0.24	0.18	0.21
$\langle \Delta E \rangle_M$	−2.98	3.41	−3.10	3.36
A^2	0.36	5.74	0.50	1.14

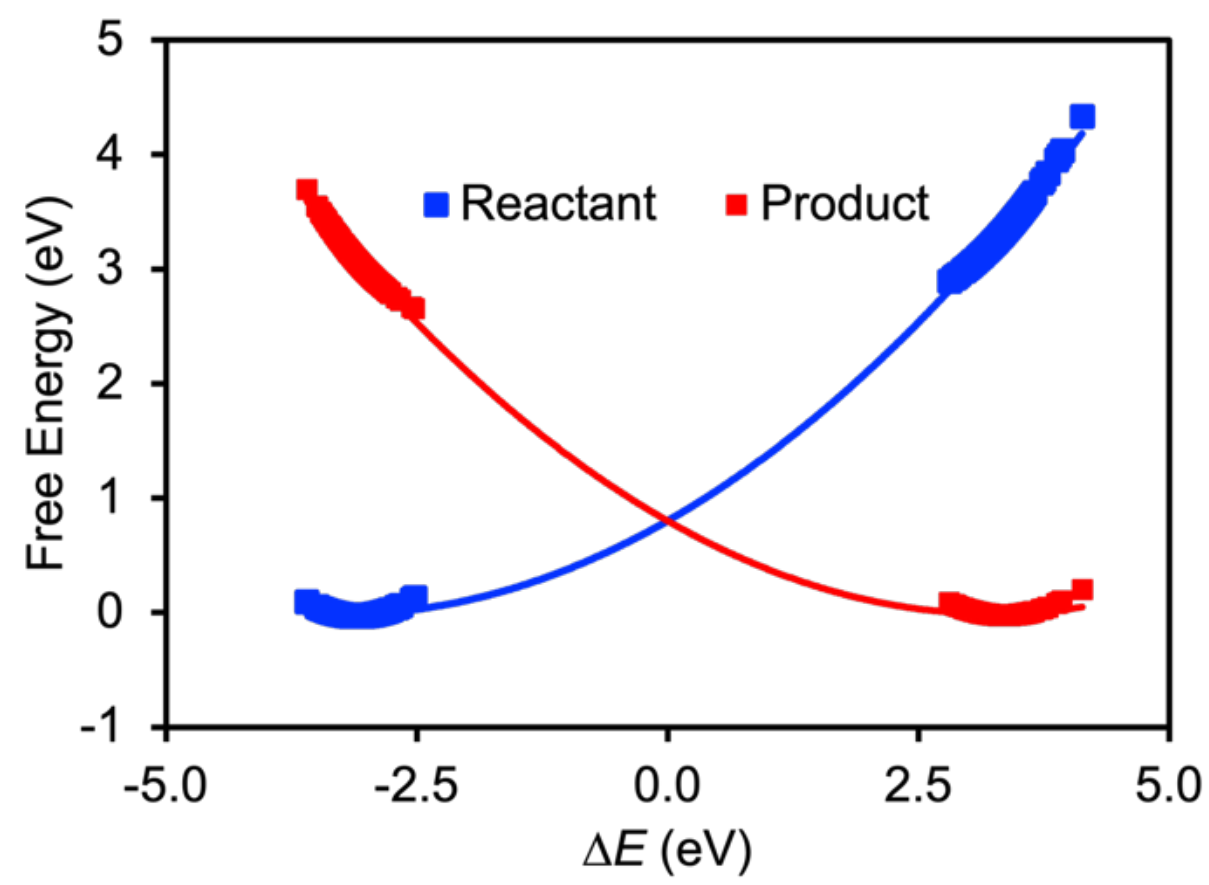

Figure S16. Free energy profiles as a function of ΔE for the Volmer step from the trajectories obtained using Nosé–Hoover thermostat at 10^{-7} Ry.

S7. Sample input files

S7.1. Optimized and starting structures to initiate AIMD

S7.1.1. Reactant

```
CELL_PARAMETERS angstrom
8.3644 0.0000 0.0000
4.1822 7.2438 0.0000
0.0000 0.0000 60.000

ATOMIC_POSITIONS angstrom
  Pt      1.3900     0.8000     -3.4000
  Pt      4.1800     0.8000     -3.4000
  Pt      6.9700     0.8000     -3.4000
  Pt      2.7900     3.2200     -3.4000
  Pt      5.5800     3.2200     -3.4000
```

Pt	8.3600	3.2200	-3.4000
Pt	4.1800	5.6300	-3.4000
Pt	6.9700	5.6300	-3.4000
Pt	9.7600	5.6300	-3.4000
Pt	0.0000	1.6100	-1.1200
Pt	2.7900	1.6100	-1.1200
Pt	5.5800	1.6100	-1.1200
Pt	1.3900	4.0200	-1.1200
Pt	4.1800	4.0200	-1.1200
Pt	6.9700	4.0200	-1.1200
Pt	2.7900	6.4400	-1.1200
Pt	5.5800	6.4400	-1.1200
Pt	8.3600	6.4400	-1.1200
Pt	-0.3917	-0.321	1.1329
Pt	2.8012	0.0074	1.1126
Pt	5.4798	0.2200	1.1700
Pt	1.2772	2.3399	1.1426
Pt	4.3351	2.3917	1.0636
Pt	7.0538	2.3103	1.0336
Pt	2.9801	4.3718	1.2809
Pt	5.5454	5.0469	0.9599
Pt	8.8309	4.9506	1.0853
O	6.6921	3.9684	4.3122
H	6.6592	3.2235	5.0444
H	6.8648	3.5984	3.4499
O	2.4652	5.6725	4.2661
H	2.5297	5.4356	3.2825
H	2.3033	4.8576	4.8588
O	8.2102	8.0231	13.3354
H	8.8656	7.3601	13.0414
H	8.2338	8.0747	14.3189
O	7.2674	2.0961	6.4840
H	6.7805	1.2249	6.7113
H	8.2174	1.8517	6.4007
O	5.1324	6.2491	4.7877
H	4.2235	6.0551	4.5593
H	5.6458	5.4172	4.9003
O	12.1285	2.7179	11.4038
H	11.7035	3.5597	11.6486
H	12.2336	2.1808	12.2504

O	12.2379	0.8146	15.9733
H	11.2270	0.8188	16.0128
H	12.5918	1.6410	16.3563
O	14.0905	-1.026	12.4065
H	14.0410	-1.684	11.6589
H	15.0110	-0.581	12.3135
O	14.7397	4.0885	13.9966
H	15.2440	3.2270	13.8832
H	13.8956	3.7726	14.5151
O	3.8169	2.5091	6.5450
H	4.3709	1.8881	6.0798
H	3.8465	2.1971	7.4963
O	6.3060	4.6447	6.9271
H	5.4744	5.0611	7.1108
H	6.1830	3.7019	7.0362
O	6.3747	6.3189	12.1423
H	6.9009	6.9790	12.5746
H	6.5255	5.4925	12.7142
O	7.9792	5.2630	16.2863
H	8.2593	6.2176	16.3029
H	7.4346	5.1222	15.4495
O	12.9002	-4.085	18.1983
H	12.1544	-4.526	18.5719
H	12.6260	-3.325	17.6193
O	9.7070	4.3034	14.3803
H	9.9445	5.1383	13.8652
H	9.1389	4.5557	15.1245
O	14.1975	3.7126	10.1539
H	13.5837	3.1857	10.6825
H	14.5557	4.4737	10.7425
O	9.6202	1.1634	16.1934
H	9.3787	1.9249	16.7576
H	9.1611	1.3970	15.2784
O	7.7653	-0.923	17.0279
H	7.0785	-0.600	17.6407
H	8.4986	-0.274	17.0260
O	11.8638	1.5294	8.9230
H	11.6346	2.1016	9.6671
H	12.7852	1.1466	9.0356
O	8.0088	1.9569	10.0253

H	8.1065	1.4419	9.2266
H	7.1940	2.5486	9.8607
O	8.2233	2.0090	13.9162
H	8.8730	2.7108	13.7284
H	8.4114	1.2503	13.2843
O	8.0461	-0.020	11.9094
H	8.7927	-0.612	11.6692
H	8.1478	0.7062	11.2457
O	5.8778	0.2810	8.2057
H	5.2503	-0.455	7.9286
H	6.6293	-0.291	8.5562
O	5.4324	7.9336	7.7467
H	5.5831	7.4901	6.8785
H	6.2598	8.1352	8.2600
O	2.6821	0.2631	3.2322
H	3.5544	0.3308	3.7044
H	2.0050	-0.301	3.6468
O	3.8520	3.8020	15.4129
H	3.5147	4.5891	15.8944
H	3.1026	3.6299	14.8602
O	1.5832	3.6904	5.8235
H	2.2941	2.9960	5.9738
H	0.7970	3.2332	5.3668
O	8.7661	5.0970	7.9632
H	7.7781	4.9961	7.9320
H	9.0290	4.4857	7.2300
O	3.8841	5.9227	8.4582
H	4.5661	6.6322	8.4206
H	4.2921	5.2870	9.0758
O	9.2339	8.0640	4.3366
H	9.6905	8.5246	3.5999
H	9.7395	7.2512	4.4063
O	5.7790	-3.223	10.5161
H	5.5541	-2.787	9.6448
H	5.1720	-4.004	10.5241

S7.1.2. Product

CELL_PARAMETERS (angstrom)
8.3644 0.0000 0.0000

4.1822 7.2438 0.0000
0.0000 0.0000 60.000

ATOMIC_POSITIONS (angstrom)

Pt	1.3900	0.8000	-3.4000
Pt	4.1800	0.8000	-3.4000
Pt	6.9700	0.8000	-3.4000
Pt	2.7900	3.2200	-3.4000
Pt	5.5800	3.2200	-3.4000
Pt	8.3600	3.2200	-3.4000
Pt	4.1800	5.6300	-3.4000
Pt	6.9700	5.6300	-3.4000
Pt	9.7600	5.6300	-3.4000
Pt	0.0000	1.6100	-1.1200
Pt	2.7900	1.6100	-1.1200
Pt	5.5800	1.6100	-1.1200
Pt	1.3900	4.0200	-1.1200
Pt	4.1800	4.0200	-1.1200
Pt	6.9700	4.0200	-1.1200
Pt	2.7900	6.4400	-1.1200
Pt	5.5800	6.4400	-1.1200
Pt	8.3600	6.4400	-1.1200
Pt	-0.0008	0.0073	1.1799
Pt	2.7779	0.0128	1.2215
Pt	5.5762	-0.006	1.1488
Pt	1.3916	2.4188	1.1788
Pt	4.1529	2.4068	1.2045
Pt	7.0068	2.4029	1.2090
Pt	2.7770	4.8459	1.1520
Pt	5.5801	4.8820	1.1957
Pt	8.3726	4.8473	1.1836
O	1.1425	5.5638	4.1772
H	2.0290	5.1234	4.0450
H	0.8696	5.2028	5.0751
O	2.8062	9.4437	11.8899
H	3.7089	9.3664	12.3118
H	2.2208	9.9626	12.5117
O	6.3104	2.6579	6.1282
H	6.2472	3.5910	5.7817
H	6.0166	2.0238	5.4056

O	5.6992	5.5315	5.8009
H	4.8182	5.6152	6.2673
H	6.3186	5.6396	6.6003
O	6.1108	4.3188	10.1126
H	5.2116	3.9129	10.1335
H	6.6682	3.6717	10.5889
O	7.5727	3.9025	16.1397
H	6.6615	3.5252	16.0740
H	8.1512	3.1108	16.2168
O	9.4679	1.6080	12.8759
H	9.8122	1.2306	12.0464
H	9.8978	2.4876	13.0344
O	11.1270	3.7400	16.3134
H	10.7410	3.9360	15.4015
H	10.5306	3.0220	16.6313
O	1.7275	2.0106	6.3250
H	1.8198	1.4504	5.5017
H	1.0772	1.5823	6.9438
O	1.2302	3.6037	9.8991
H	1.2284	4.4568	9.4364
H	0.7239	2.9825	9.3080
O	2.1826	6.7539	12.1275
H	2.3642	7.7282	12.1593
H	1.2563	6.6072	12.4137
O	3.7492	6.3867	16.0636
H	2.8175	6.7243	15.9114
H	3.6067	5.5063	16.5094
O	9.3524	1.5592	16.0609
H	8.9092	0.7239	16.3359
H	9.4981	1.4746	15.0908
O	5.4229	6.0612	13.8998
H	5.2584	6.9101	13.4376
H	4.8349	6.1208	14.7148
O	10.1996	4.1010	13.7227
H	9.3878	4.6703	13.7237
H	10.8476	4.6477	13.2562
O	5.0614	2.5245	15.6245
H	4.2258	2.9026	16.0208
H	4.9901	2.8044	14.6752
O	5.3037	-0.076	15.8204

H	4.8208	-0.455	16.5734
H	5.1743	0.9419	15.8212
O	8.1265	1.8101	8.1863
H	7.4549	2.1373	7.5415
H	7.6867	1.0863	8.7070
O	4.0156	2.7299	7.5358
H	4.8302	2.7257	6.9566
H	3.2317	2.4100	6.9971
O	4.9899	3.5238	13.1231
H	5.1004	4.5236	13.2427
H	4.4153	3.3635	12.3284
O	3.7485	2.9369	10.7765
H	3.9060	2.0216	10.4187
H	2.8344	3.1325	10.4381
O	4.4013	0.6425	9.1364
H	3.7999	-0.086	8.8537
H	4.2548	1.3944	8.5096
O	3.4064	5.3574	7.3996
H	3.6903	4.4320	7.6385
H	3.2107	5.8371	8.2509
O	2.3621	0.4466	4.1436
H	3.3504	0.5421	4.2353
H	2.1520	-0.438	4.5902
O	-0.0374	6.0119	13.6493
H	0.3065	6.4720	14.4641
H	-0.9982	5.9026	13.8208
O	0.9591	4.5907	6.6465
H	1.8776	4.9537	6.9000
H	1.1377	3.6112	6.4787
O	7.2723	5.6370	7.9869
H	6.8656	5.0713	8.6976
H	8.0733	5.1633	7.6074
O	2.6278	6.8610	9.4909
H	2.4841	6.7010	10.4775
H	1.7124	7.2806	9.2741
O	9.3744	8.1552	4.3620
H	9.5501	8.4163	3.4274
H	9.5512	7.1432	4.3160
O	1.4010	-0.074	10.6731
H	0.5087	-0.073	10.2842

H	1.6968	-1.0185	10.6650
O	3.5204	4.1098	4.2716
H	4.3690	4.5597	4.4173
H	3.6287	3.5166	3.4948
H	5.5768	3.2328	2.0842

S7.2. Geometry optimization input file with ASE

```
from ase.io import read, write
from ase.calculators.espresso import Espresso, EspressoProfile
from ase.optimize import BFGS
from ase.constraints import ExpCellFilter
from ase.constraints import FixAtoms
import os

jobname = 'pt111sol'
pseudos={"Pt":"Pt.pbe-n-rrkjus_psl.1.0.0.UPF",
        "O":"O.pbe-n-rrkjus_psl.1.0.0.UPF",
        "H":"H.pbe-rrkjus_psl.1.0.0.UPF"}

myaseobj=read(jobname + '.POSCAR')

# Set QE pw.x command
pwx_command = "put the command"

# Constrain all atoms below a z-position
c = FixAtoms(indices=[myaseobj.index for myaseobj in myaseobj if
myaseobj.position[2] < -0.2])
myaseobj.set_constraint(c)

# Calculation parameters
calc = Espresso(profile = EspressoProfile(
                command = pwx_command,
                pseudo_dir = 'path of the pseudopotential directroy'),
                title = jobname, prefix = jobname,
                outdir = './',
                conv_thr = 1e-6,
                ecutwfc = 40,
                ecutrho = 410,
                startingwfc = 'atomic',
                startingpot = 'atomic',
```

```
                    nspin = 1,
                    input_dft = 'pbe',
                    vdw_corr = 'dft-d3',
                    occupations = 'smearing',
                    smearing = 'gaussian',
                    degauss = 0.01,
                    nosym = True,
                    mixing_beta = 0.1,
                    mixing_ndim = 12,
                    pseudo_dir = 'path of the pseudopotential directory',
                    pseudopotentials=pseudos,
                    tprnfor=True,
                    electron_maxstep = 399,
                    tot_charge = 0,
                    assume_isolated = 'esm',
                    esm_bc = 'bc3',
                    esm_w = 0.1,
                    kpts=(2,2,1))

# Run the calculation
myaseobj.calc = calc
bfgs = BFGS(myaseobj, trajectory = jobname + '.traj', logfile = jobname + '.log')
bfgs.run(fmax=0.05)
myaseobj.get_potential_energy()

# Write optimized geometry to file
NewPOSCAR= read(jobname + '.traj')
write(jobname + '.opt.POSCAR',NewPOSCAR)
```

S7.3. Single point calculation input file

```
from ase.io import read, write
from ase.calculators.espresso import Espresso, EspressoProfile
from ase.optimize import BFGS
from ase.constraints import ExpCellFilter
from ase.constraints import FixAtoms
import os

jobname = 'reactant'
pseudos={"Pt":"Pt.pbe-n-rrkjus_psl.1.0.0.UPF",
```

```
        "O":"O.pbe-n-rrkjus_psl.1.0.0.UPF",
        "H":"H.pbe-rrkjus_psl.1.0.0.UPF"}

myaseobj=read(jobname + '.POSCAR')

# Set QE pw.x command
pwx_command = "put the command"

# Constrain all atoms below a z-position
c = FixAtoms(indices=[myaseobj.index for myaseobj in myaseobj if
myaseobj.position[2] < -0.2])
myaseobj.set_constraint([c])

# make hydrogens deuterium
tfmat = [myaseobj.index for myaseobj in myaseobj if myaseobj.symbol == 'H']
masses = myaseobj.get_masses()
for n in tfmat:
    masses[n] = 2
myaseobj.set_masses(masses)

# Calculation parameters
calc = Espresso(profile = EspressoProfile(
                command = pwx_command,
                pseudo_dir = 'path of the pseudopotential directroy'),
                title = jobname, prefix = jobname,
                outdir = './',
                conv_thr = 1e-6,
                ecutwfc = 40,
                ecutrho = 410,
                startingwfc = 'atomic',
                startingpot = 'atomic',
                nspin = 1,
                input_dft = 'pbe',
                vdw_corr = 'dft-d3',
                occupations = 'smearing',
                smearing = 'gaussian',
                degauss = 0.01,
                nosym = True,
                mixing_beta = 0.1,
                mixing_ndim = 12,
```

```
        pseudo_dir = 'path of the pseudopotential directory',
        pseudopotentials=pseudos,
        tprnfor=True,
        electron_maxstep = 399,
        assume_isolated = 'esm',
        lgcscf = True,
        esm_bc = 'bc3',
        esm_w = 0.1,
        gcscf_mu = -5.0,
        kpts=(2,2,1))

# Run the calculation
myaseobj.calc = calc
myaseobj.get_potential_energy()
```

S7.4. AIMD input file (Berendsen dynamics in Quantum ESPRESSO)

```
&control
   calculation = 'md'
   pseudo_dir = 'path of the pseudopotential directory'
   outdir = './'
   title = 'pt.h2o'
   prefix = 'pt.h2o'
   forc_conv_thr = 1D-3
   nstep = 100000
   iprint = 1
   dt = 20.67,
   wf_collect = .true.
   max_seconds = 86000
/
&SYSTEM
  tot_charge       = 0
  ecutwfc          = 40
  ecutrho          = 410
  nosym            = .true.
  occupations      = 'smearing'
  degauss          = 0.01
  smearing         = 'gaussian'
  nspin            = 1
  input_dft        = 'pbe'
  assume_isolated  = 'esm'
```

```
  esm_bc          = 'bc3'
  esm_w           = 0.1
  vdw_corr        = 'grimme-d3'
  ntyp            = 3
  nat             = 120
  ibrav           = 0
/
&electrons
   startingpot = 'file'
   startingwfc = 'file'
   mixing_mode = 'plain'
   mixing_ndim = 12
   mixing_beta = 0.1,
   electron_maxstep=399,
   conv_thr =  1.0d-4
   diagonalization = 'rmm-davidson'
/
&ions
   ion_dynamics = 'verlet'
   pot_extrapolation = 'none'
   wfc_extrapolation = 'none'
   ion_temperature = 'berendsen'
   tempw = 350.D0,
/
&CELL
/
&FCP
/
&RISM
/
ATOMIC_SPECIES
Pt 195.084 Pt.pbe-n-rrkjus_psl.1.0.0.UPF
H 2.0 H.pbe-rrkjus_psl.1.0.0.UPF
O 15.999 O.pbe-n-rrkjus_psl.1.0.0.UPF

K_POINTS gamma
```

S7.5. AIMD input file (Nosé–Hoover in Quantum ESPRESSO)

```
&control
   calculation = 'md'
```

```
    ! restart_mode = 'restart'
    pseudo_dir = 'path of the pseudopotential directory'
    outdir = './'
    title = 'pt.h2o'
    prefix = 'pt.h2o'
    forc_conv_thr = 1D-3
    nstep = 100000
    iprint = 1
    dt = 20.67,
    wf_collect = .true.
    max_seconds = 172000
/
&SYSTEM
   tot_charge      = 0
   ecutwfc         = 40
   ecutrho         = 410
   nosym           = .true.
   occupations     = 'smearing'
   degauss         = 0.01
   smearing        = 'gaussian'
   nspin           = 1
   input_dft       = 'pbe'
   assume_isolated  = 'esm'
   esm_bc          = 'bc3'
   esm_w           = 0.1
   vdw_corr        = 'grimme-d3'
   ntyp            = 3
   nat             = 120
   ibrav           = 0
/
&electrons
    startingpot = 'file'
    startingwfc = 'file'
    mixing_mode = 'plain'
    mixing_ndim = 12
    mixing_beta = 0.1,
    electron_maxstep=2000,
    conv_thr =  1.0d-4
    diagonalization = 'rmm-davidson'
/
```

```
&ions
   ion_dynamics = 'verlet'
   pot_extrapolation = 'none'
   wfc_extrapolation = 'none'
   ion_temperature = 'nose'
   tempw = 350.D0,
   fnosep = 20
   nhpcl = 3
/
&CELL
/
&FCP
/
&RISM
/
ATOMIC_SPECIES
Pt 195.084 Pt.pbe-n-rrkjus_psl.1.0.0.UPF
H 2.0 H.pbe-rrkjus_psl.1.0.0.UPF
O 15.999 O.pbe-n-rrkjus_psl.1.0.0.UPF

K_POINTS gamma
```

S7.6. AIMD input file (Langevin dynamics with ASE)

```
from ase.io import read, write
from ase.calculators.espresso import Espresso, EspressoProfile
from ase.constraints import FixAtoms
from ase.md import MDLogger
from ase.md.langevin import Langevin
from ase.units import Bohr,Rydberg,kJ,kB,fs,Hartree,mol,kcal
import os

jobname = 'pt111sol'
pseudos={"Pt":"Pt.pbe-n-rrkjus_psl.1.0.0.UPF",
        "O":"O.pbe-n-rrkjus_psl.1.0.0.UPF",
        "H":"H.pbe-rrkjus_psl.1.0.0.UPF"}

myaseobj=read(jobname + '.POSCAR')

# Set QE pw.x command
pwx_command = "command"
```

```
# Constrain all atoms below a z-position of -0.2 Å
c = FixAtoms(indices=[myaseobj.index for myaseobj in myaseobj if
myaseobj.position[2] < -0.2])
myaseobj.set_constraint(c)

# make hydrogens deuterium
tfmat = [myaseobj.index for myaseobj in myaseobj if myaseobj.symbol == 'H']
masses = myaseobj.get_masses()
for n in tfmat:
   masses[n] = 2
myaseobj.set_masses(masses)

# Calculation parameters
calc = Espresso(profile = EspressoProfile(
            command = pwx_command,
            pseudo_dir = 'path of the pseudopotential directory'),
            title = jobname, prefix = jobname,
            outdir = './',
            conv_thr = 1e-4,
            ecutwfc = 40,
            ecutrho = 410,
            startingwfc = 'file',
            startingpot = 'file',
            nspin = 1,
            input_dft = 'pbe',
            vdw_corr = 'dft-d3',
            occupations = 'smearing',
            smearing = 'gaussian',
            degauss = 0.01,
            nosym = True,
            mixing_beta = 0.1,
            mixing_ndim = 2,
            pseudo_dir = 'path of the pseudopotential directory',
            pseudopotentials=pseudos,
            tprnfor=True,
            electron_maxstep = 399,
            tot_charge = 0,
            assume_isolated = 'esm',
            esm_bc = 'bc3',
```

```
        esm_w = 0.1,
        kpts=(1,1,1))

# Run the calculation
myaseobj.calc = calc

#Langevin Dynamics setup
dyn = Langevin(
  myaseobj,
  timestep=1.0 * fs,
  fixcm = False,
  temperature_K=350.0,
  friction=0.01 / fs,
  trajectory=jobname + '.traj',
  logfile=jobname + '.log')

# Run the dynamics
dyn.run(1000)
```

References

(1) Giannozzi, P.; Baroni, S.; Bonini, N.; Calandra, M.; Car, R.; Cavazzoni, C.; Ceresoli, D.; Chiarotti, G. L.; Cococcioni, M.; Dabo, I.; Dal Corso, A.; De Gironcoli, S.; Fabris, S.; Fratesi, G.; Gebauer, R.; Gerstmann, U.; Gougoussis, C.; Kokalj, A.; Lazzeri, M.; Martin-Samos, L.; Marzari, N.; Mauri, F.; Mazzarello, R.; Paolini, S.; Pasquarello, A.; Paulatto, L.; Sbraccia, C.; Scandolo, S.; Sclauzero, G.; Seitsonen, A. P.; Smogunov, A.; Umari, P.; Wentzcovitch, R. M. QUANTUM ESPRESSO: A Modular and Open-Source Software Project for Quantum Simulations of Materials. *J. Phys. Condens. Matter* **2009**, *21*, 395502.

(2) Giannozzi, P.; Andreussi, O.; Brumme, T.; Bunau, O.; Buongiorno Nardelli, M.; Calandra, M.; Car, R.; Cavazzoni, C.; Ceresoli, D.; Cococcioni, M.; Colonna, N.; Carnimeo, I.; Dal Corso, A.; De Gironcoli, S.; Delugas, P.; DiStasio, R. A.; Ferretti, A.; Floris, A.; Fratesi, G.; Fugallo, G.; Gebauer, R.; Gerstmann, U.; Giustino, F.; Gorni, T.; Jia, J.; Kawamura, M.; Ko, H.-Y.; Kokalj, A.; Küçükbenli, E.; Lazzeri, M.; Marsili, M.; Marzari, N.; Mauri, F.; Nguyen, N. L.; Nguyen, H.-V.; Otero-de-la-Roza, A.; Paulatto, L.; Poncé, S.; Rocca, D.; Sabatini, R.; Santra, B.; Schlipf, M.; Seitsonen, A. P.; Smogunov, A.; Timrov, I.; Thonhauser, T.; Umari, P.; Vast, N.; Wu, X.; Baroni, S. Advanced Capabilities for Materials Modelling with Quantum ESPRESSO. *J. Phys. Condens. Matter* **2017**, *29*, 465901.

(3) Hjorth Larsen, A.; Jørgen Mortensen, J.; Blomqvist, J.; Castelli, I. E.; Christensen, R.; Dułak, M.; Friis, J.; Groves, M. N.; Hammer, B.; Hargus, C.; Hermes, E. D.; Jennings, P. C.; Bjerre Jensen, P.; Kermode, J.; Kitchin, J. R.; Leonhard Kolsbjerg, E.; Kubal, J.; Kaasbjerg, K.; Lysgaard, S.; Bergmann Maronsson, J.; Maxson, T.; Olsen, T.; Pastewka, L.; Peterson, A.; Rostgaard, C.; Schiøtz, J.; Schütt, O.; Strange, M.; Thygesen, K. S.; Vegge, T.; Vilhelmsen, L.; Walter, M.; Zeng, Z.; Jacobsen, K. W. The Atomic Simulation Environment—a Python Library for Working with Atoms. *J. Phys. Condens. Matter* **2017**, *29*, 273002.

(4) Perdew, J. P.; Burke, K.; Ernzerhof, M. Generalized Gradient Approximation Made Simple. *Phys. Rev. Lett.* **1996**, *77*, 3865–3868.

(5) Grimme, S.; Antony, J.; Ehrlich, S.; Krieg, H. A Consistent and Accurate *Ab Initio* Parametrization of Density Functional Dispersion Correction (DFT-D) for the 94 Elements H-Pu. *J. Chem. Phys.* **2010**, *132*, 154104.

(6) Vanderbilt, D. Soft Self-Consistent Pseudopotentials in a Generalized Eigenvalue Formalism. *Phys. Rev. B* **1990**, *41*, 7892–7895.

(7) Sundararaman, R.; Goddard, W. A.; Arias, T. A. Grand Canonical Electronic Density-Functional Theory: Algorithms and Applications to Electrochemistry. *J. Chem. Phys.* **2017**, *146*, 114104.

(8) Rossmeisl, J.; Skúlason, E.; Björketun, M. E.; Tripkovic, V.; Nørskov, J. K. Modeling the Electrified Solid–Liquid Interface. *Chem. Phys. Lett.* **2008**, *466*, 68–71.

(9) Chan, K.; Nørskov, J. K. Electrochemical Barriers Made Simple. *J. Phys. Chem. Lett.* **2015**, *6*, 2663–2668.

(10) Chan, K.; Nørskov, J. K. Potential Dependence of Electrochemical Barriers from Ab Initio Calculations. *J. Phys. Chem. Lett.* **2016**, *7*, 1686–1690.

(11) Agrawal, N.; Wong, A. J.-W.; Maheshwari, S.; Janik, M. J. An Efficient Approach to Compartmentalize Double Layer Effects on Kinetics of Interfacial Proton-Electron Transfer Reactions. *J. Catal.* **2024**, *430*, 115360.

(12) Hörmann, N. G.; Beinlich, S. D.; Reuter, K. Converging Divergent Paths: Constant Charge vs Constant Potential Energetics in Computational Electrochemistry. *J. Phys. Chem. C* **2024**, *128*, 5524–5531.
(13) Kastlunger, G.; Vijay, S.; Chen, X.; Sharma, S.; Peterson, A. On the Thermodynamic Equivalence of Grand Canonical, Infinite-Size, and Capacitor-Based Models in First-Principle Electrochemistry. *ChemPhysChem* **2024**, *25*, e202300950.
(14) Berendsen, H. J. C.; Postma, J. P. M.; Van Gunsteren, W. F.; DiNola, A.; Haak, J. R. Molecular Dynamics with Coupling to an External Bath. *J. Chem. Phys.* **1984**, *81*, 3684–3690.
(15) Evans, D. J.; Holian, B. L. The Nose–Hoover Thermostat. *J. Chem. Phys.* **1985**, *83*, 4069–4074.
(16) Vanden-Eijnden, E.; Ciccotti, G. Second-Order Integrators for Langevin Equations with Holonomic Constraints. *Chem. Phys. Lett.* **2006**, *429*, 310–316.
(17) Otani, M.; Sugino, O. First-Principles Calculations of Charged Surfaces and Interfaces: A Plane-Wave Nonrepeated Slab Approach. *Phys. Rev. B* **2006**, *73*, 115407.
(18) Mahoney, M. W.; Jorgensen, W. L. A Five-Site Model for Liquid Water and the Reproduction of the Density Anomaly by Rigid, Nonpolarizable Potential Functions. *J. Chem. Phys.* **2000**, *112*, 8910–8922.
(19) Abraham, M. J.; Murtola, T.; Schulz, R.; Páll, S.; Smith, J. C.; Hess, B.; Lindahl, E. GROMACS: High Performance Molecular Simulations through Multi-Level Parallelism from Laptops to Supercomputers. *SoftwareX* **2015**, *1–2*, 19–25.
(20) Hollóczki, O.; Macchiagodena, M.; Weber, H.; Thomas, M.; Brehm, M.; Stark, A.; Russina, O.; Triolo, A.; Kirchner, B. Triphilic Ionic-Liquid Mixtures: Fluorinated and Non-fluorinated Aprotic Ionic-Liquid Mixtures. *ChemPhysChem* **2015**, *16*, 3325–3333.
(21) Thomas, M.; Brehm, M.; Hollóczki, O.; Kelemen, Z.; Nyulászi, L.; Pasinszki, T.; Kirchner, B. Simulating the Vibrational Spectra of Ionic Liquid Systems: 1-Ethyl-3-Methylimidazolium Acetate and Its Mixtures. *J. Chem. Phys.* **2014**, *141*, 024510.
(22) Caro, M. A.; Laurila, T.; Lopez-Acevedo, O. Accurate Schemes for Calculation of Thermodynamic Properties of Liquid Mixtures from Molecular Dynamics Simulations. *J. Chem. Phys.* **2016**, *145*, 244504.
(23) Lin, S.-T.; Blanco, M.; Goddard, W. A. The Two-Phase Model for Calculating Thermodynamic Properties of Liquids from Molecular Dynamics: Validation for the Phase Diagram of Lennard-Jones Fluids. *J. Chem. Phys.* **2003**, *119*, 11792–11805.
(24) Chen, M.; Ko, H.-Y.; Remsing, R. C.; Calegari Andrade, M. F.; Santra, B.; Sun, Z.; Selloni, A.; Car, R.; Klein, M. L.; Perdew, J. P.; Wu, X. Ab Initio Theory and Modeling of Water. *Proc. Natl. Acad. Sci.* **2017**, *114*, 10846–10851.
(25) Zhou, K.; Qian, C.; Liu, Y. Quantifying the Structure of Water and Hydrated Monovalent Ions by Density Functional Theory-Based Molecular Dynamics. *J. Phys. Chem. B* **2022**, *126*, 10471–10480.
(26) Joutsuka, T. Molecular Mechanism of Autodissociation in Liquid Water: *Ab Initio* Molecular Dynamics Simulations. *J. Phys. Chem. B* **2022**, *126*, 4565–4571.
(27) Li, P.; Huang, J.; Hu, Y.; Chen, S. Establishment of the Potential of Zero Charge of Metals in Aqueous Solutions: Different Faces of Water Revealed by Ab Initio Molecular Dynamics Simulations. *J. Phys. Chem. C* **2021**, *125*, 3972–3979.

(28) Le, J.; Cuesta, A.; Cheng, J. The Structure of Metal-Water Interface at the Potential of Zero Charge from Density Functional Theory-Based Molecular Dynamics. *J. Electroanal. Chem.* **2018**, *819*, 87–94.
(29) Sakong, S.; Groß, A. Water Structures on a Pt(111) Electrode from *Ab Initio* Molecular Dynamic Simulations for a Variety of Electrochemical Conditions. *Phys. Chem. Chem. Phys.* **2020**, *22*, 10431–10437.
(30) Tesch, R.; Kowalski, P. M.; Eikerling, M. H. Properties of the Pt(111)/Electrolyte Electrochemical Interface Studied with a Hybrid DFT–Solvation Approach. *J. Phys. Condens. Matter* **2021**, *33*, 444004.
(31) Le, J.; Fan, Q.; Perez-Martinez, L.; Cuesta, A.; Cheng, J. Theoretical Insight into the Vibrational Spectra of Metal–Water Interfaces from Density Functional Theory Based Molecular Dynamics. *Phys. Chem. Chem. Phys.* **2018**, *20*, 11554–11558.
(32) Goldsmith, Z. K.; Calegari Andrade, M. F.; Selloni, A. Effects of Applied Voltage on Water at a Gold Electrode Interface from *Ab Initio* Molecular Dynamics. *Chem. Sci.* **2021**, *12*, 5865–5873.
(33) Korpelin, V.; Kiljunen, T.; Melander, M. M.; Caro, M. A.; Kristoffersen, H. H.; Mammen, N.; Apaja, V.; Honkala, K. Addressing Dynamics at Catalytic Heterogeneous Interfaces with DFT-MD: Anomalous Temperature Distributions from Commonly Used Thermostats. *J. Phys. Chem. Lett.* **2022**, *13*, 2644–2652.
(34) Sun, Q.; Oliveira, N. J.; Kwon, S.; Tyukhtenko, S.; Guo, J. J.; Myrthil, N.; Lopez, S. A.; Kendrick, I.; Mukerjee, S.; Ma, L.; Ehrlich, S. N.; Li, J.; Goddard, W. A.; Yan, Y.; Jia, Q. Understanding Hydrogen Electrocatalysis by Probing the Hydrogen-Bond Network of Water at the Electrified Pt–Solution Interface. *Nat. Energy* **2023**, *8*, 859–869.
(35) Marx, D.; Tuckerman, M. E.; Hutter, J.; Parrinello, M. The Nature of the Hydrated Excess Proton in Water. *Nature* **1999**, *397*, 601–604.
(36) Marx, D.; Tuckerman, M. E.; Parrinello, M. Solvated Excess Protons in Water: Quantum Effects on the Hydration Structure. *J. Phys. Condens. Matter* **2000**, *12*, A153–A159.
(37) Marx, D.; Chandra, A.; Tuckerman, M. E. Aqueous Basic Solutions: Hydroxide Solvation, Structural Diffusion, and Comparison to the Hydrated Proton. *Chem. Rev.* **2010**, *110*, 2174–2216.
(38) Rossi, M.; Ceriotti, M.; Manolopoulos, D. E. Nuclear Quantum Effects in H^+ and OH^- Diffusion along Confined Water Wires. *J. Phys. Chem. Lett.* **2016**, *7*, 3001–3007.
